# Cervical cancer prevention and care in HIV clinics across sub‐Saharan Africa: results of a facility‐based survey

**DOI:** 10.1002/jia2.26303

**Published:** 2024-07-09

**Authors:** Serra Lem Asangbeh‐Kerman, Maša Davidović, Katayoun Taghavi, Tafadzwa Dhokotera, Albert Manasyan, Anjali Sharma, Antoine Jaquet, Beverly Musick, Christella Twizere, Cleophas Chimbetete, Gad Murenzi, Hannock Tweya, Josephine Muhairwe, Kara Wools‐Kaloustian, Karl‐Gunter Technau, Kathryn Anastos, Marcel Yotebieng, Marielle Jousse, Oliver Ezechi, Omenge Orang'o, Samuel Bosomprah, Simon Pierre Boni, Partha Basu, Julia Bohlius, IeDEA

**Affiliations:** ^1^ Graduate School of Cellular and Biomedical Sciences University of Bern Bern Switzerland; ^2^ Swiss Tropical and Public Health Institute Allschwil Switzerland; ^3^ University of Basel Basel Switzerland; ^4^ Graduate School for Health Sciences University of Bern Bern Switzerland; ^5^ Institute of Social and Preventive Medicine University of Bern Bern Switzerland; ^6^ Centre for Infectious Disease Research in Zambia Lusaka Zambia; ^7^ Division of Neonatology Department of Pediatrics University of Alabama at Birmingham Birmingham Alabama USA; ^8^ University of Bordeaux National Institute for Health and Medical Research (INSERM) UMR 1219 Research Institute for Sustainable Development (IRD) EMR 271 Bordeaux Population Health Centre Bordeaux France; ^9^ Department of Biostatistics and Health Data Science School of Medicine Indiana University Indianapolis Indiana USA; ^10^ Centre National de Reference en Matière de VIH/SIDA Bujumbura Burundi; ^11^ Newlands Clinic Harare Zimbabwe; ^12^ Einstein‐Rwanda Research and Capacity Building Programme Research for Development and Rwanda Military Hospital Kigali Rwanda; ^13^ International Training and Education Centre for Health (I‐TECH) Lilongwe Malawi; ^14^ SolidarMed Partnerships for Health Maseru Lesotho; ^15^ Institute of Global Health University of Geneva Geneva Switzerland; ^16^ Department of Medicine Indiana University School of Medicine Indianapolis Indiana USA; ^17^ Empilweni Services and Research Unit, Rahima Moosa Mother and Child Hospital Faculty of Health Sciences University of the Witwatersrand Johannesburg South Africa; ^18^ Department of Paediatrics and Child Health Rahima Moosa Mother and Child Hospital Johannesburg‐Braamfontein South Africa; ^19^ Department of Medicine and Epidemiology Albert Einstein College of Medicine Bronx New York USA; ^20^ Department of Medicine Albert Einstein College of Medicine Bronx New York USA; ^21^ SolidarMed, Partnership for Health Chiure Mozambique; ^22^ Department of Clinical Sciences Nigerian Institute of Medical Research Lagos Nigeria; ^23^ Moi University Eldoret Kenya; ^24^ Department of Biostatistics School of Public Health University of Ghana Accra Ghana; ^25^ Programme National de Lutte contre le Cancer (PNLCa) Abidjan Côte d'Ivoire; ^26^ Programme PAC‐CI Site ANRS Treichville Abidjan Côte d'Ivoire; ^27^ Early Detection, Prevention and Infections Branch, International Agency for Research on Cancer Lyon France

**Keywords:** cervical cancer prevention, HIV, monitoring, outcomes, prevention and care cascades, sub‐Saharan Africa

## Abstract

**INTRODUCTION:**

To eliminate cervical cancer (CC), access to and quality of prevention and care services must be monitored, particularly for women living with HIV (WLHIV). We assessed implementation practices in HIV clinics across sub‐Saharan Africa (SSA) to identify gaps in the care cascade and used aggregated patient data to populate cascades for WLHIV attending HIV clinics.

**METHODS:**

Our facility‐based survey was administered between November 2020 and July 2021 in 30 HIV clinics across SSA that participate in the International epidemiology Databases to Evaluate AIDS (IeDEA) consortium. We performed a qualitative site‐level assessment of CC prevention and care services and analysed data from routine care of WLHIV in SSA.

**RESULTS:**

Human papillomavirus (HPV) vaccination was offered in 33% of sites. Referral for CC diagnosis (42%) and treatment (70%) was common, but not free at about 50% of sites. Most sites had electronic health information systems (90%), but data to inform indicators to monitor global targets for CC elimination in WLHIV were not routinely collected in these sites. Data were collected routinely in only 36% of sites that offered HPV vaccination, 33% of sites that offered cervical screening and 20% of sites that offered pre‐cancer and CC treatment.

**CONCLUSIONS:**

Though CC prevention and care services have long been available in some HIV clinics across SSA, patient and programme monitoring need to be improved. Countries should consider leveraging their existing health information systems and use monitoring tools provided by the World Health Organization to improve CC prevention programmes and access, and to track their progress towards the goal of eliminating CC.

## 1 INTRODUCTION

The World Health Organization (WHO) seeks to eliminate cervical cancer (CC) within this century, and has defined the “90‐70‐90” targets it expects countries to reach by 2030: 90% of girls must be vaccinated with an HPV vaccine by the time they are 15 years old; 70% of women screened with a high‐performance test at 35 and 45 years; and 90% of women diagnosed with cervical pre‐cancer or cancer should be treated [1]. To achieve these targets, countries that have a high HIV burden must adopt CC prevention strategies that meet the specific needs of girls and women living with HIV (WLHIV), since they are more susceptible to disease than HIV‐negative girls and women [2, 3]. This requires health sector reform to deliver comprehensive prevention and care services, including expanding community awareness, biomedical and clinical interventions, improving quality assurance and monitoring mechanisms, and providing the financial and technical resources necessary to implement programmes [4, 5].

When nations implement preventive HIV and CC services that meet women's needs over time and across different levels of their health systems, uptake of screening services and clinical outcomes both improve. This integrated service delivery model has been adopted by several sub‐Saharan African countries that have a high HIV burden [6, 7, 8, 9, 10, 11, 12]. But these programmes are too often opportunistic with low coverage, so gains in reducing CC incidence and mortality may wane over time. Permanent reduction in CC incidence and mortality like in high‐income countries [13] must be monitored using routinely collected data that informs indicators. Without such data, countries cannot assess their progress, identify gaps and devise effective interventions against CC [1].

Previous studies reported pre‐cancer treatment rates of 25.6% in WLHIV in a public hospital in South Africa, 76.2% in women regardless of HIV status in Zambia and 78% in WLHIV in one clinic in Zimbabwe [14, 15, 16]. A 2017 systematic review suggested an extension of screening options applied to HIV‐negative women, to WLHIV, with more frequent follow‐up [2]. These studies do not report on all three WHO elimination targets or on other aspects of a comprehensive CC prevention and control programme. CC prevention practices within HIV clinics are rarely described and facilities rarely report data necessary to monitor WHO targets for eliminating CC in WLHIV.

We set out to fill these gaps with a survey‐based study to qualitatively assess the implementation of CC prevention services across sub‐Saharan Africa (SSA) at the facility/site level and use aggregate patient data to quantitatively assess cascades for WLHIV attending HIV clinics with fairly advanced CC prevention programmes.

## 2 METHODS

### 2.1 Study design and setting

We conducted this facility‐based survey between November 2020 and July 2021 at 30 HIV clinics in four African regions that participate in the International Epidemiology Databases to Evaluate AIDS (IeDEA) consortium. IeDEA is a global network that gathers and analyses routinely collected clinical data from children, adolescents and adults living with HIV across 240 HIV treatment and care sites (https://www.iedea.org/). The IeDEA regional principal investigators for central, East, southern and West Africa did a convenience sampling of 30 HIV clinics that offered CC prevention and control services on‐ or off‐site, and had electronic or paper‐based systems for data collection.

### 2.2 Study participants

We collected data for HPV vaccination and cervical screening in the following four populations:

- HPV vaccination
  - *Girls, adolescents/young WLHIV in care*: girls aged 9–14 years and/or adolescents or young women aged 15–26 years who had at least one HIV medical care visit in the clinic during the index year (the year for which data were reported);
  - *Girls and/or adolescents and young WLHIV eligible for HPV vaccination*: according to each site's eligibility criteria.
- Cervical screening
  - *WLHIV in care*: 15 years old or older, who had at least one HIV medical care visit during the index year;
  - *WLHIV eligible for cervical screening*: according to each site's eligibility criteria.

We harmonized these definitions of girls and women in care to ensure data could be compared across sites in different countries.

### 2.3 Survey development

We constructed a survey, which we based on both the International Agency for Research on Cancer CANscreen5 tool (https://canscreen5.iarc.fr/) and the WHO Toolkit for Cervical Cancer prevention and control programmes [17]. First, we organized a meeting with IeDEA principal investigators, data managers, and the CANscreen5 and WHO toolkit development team members to discuss the scope of the study, study population, site eligibility and index years for data collection. Second, the lead author (SLA‐K) visited six participating sites to discuss the survey with programme teams, then revised it based on their input. The revised survey was programmed into Research Electronic Data Capture (REDCap 9.8.2), a web‐based application used to create databases and projects. We offered the survey in English and French.

We qualitatively assessed CC prevention and care services across six domains: (1) respondent and site characteristics; (2) HPV vaccination; (3) CC screening, diagnosis, and treatment; (4) data collection and aggregation systems; (5) evaluations and audits; and (6) decision and referral support systems.

We analysed aggregated data routinely collected for HPV vaccination, cervical screening, diagnosis and treatment services offered to WLHIV in these sites. We prioritized the WHO global indicators [17] that had been reported and included HPV vaccination proportion (a key indicator in monitoring WHO targets for eliminating CC).

### 2.4 Survey piloting and data collection

Between May and August 2020, we piloted the survey at two sites, one in West and one in East Africa, collected feedback and then revised the survey. Target respondents were CC prevention and control programme managers or health personnel involved in CC screening activities. We invited respondents via an email that included automatically generated links to the survey. Sites that had challenges using REDCap 9.8.2 printed the forms, filled them in by hand and submitted scanned copies through a secured email server. One researcher (SLA‐K) manually entered scanned responses into REDCap 9.8.2 and another (MD) checked the entries. Site investigators could also check the accuracy of their site data and could query the lead author if they detected any problems.

### 2.5 Statistical analyses

The primary outcomes of interest for our analysis were: the availability and use of CC prevention services; and the proportion of girls vaccinated, women screened, and/or treated for cervical pre‐cancer and CC. We used descriptive statistics to report site characteristics and calculated percentages for each indicator. We used a changing denominator (target approach) to calculate the CC prevention and care cascade, in which all women who reach a given step comprise the denominator for each subsequent step. The target approach highlights retention gaps where they appear in cascades [18]. We assessed the association of facility characteristics (facility location, facility type, services integration, presence of non‐governmental organization [NGO] support for CC prevention) and availability of patient‐level data to inform key performance indicators using chi‐square and Fischer's tests as appropriate. We reported outcomes for sites with data disaggregated by HIV status, if they included data for 10 or more eligible girls or women in care. We chose this low cut‐off because many sites (especially sites that vaccinated girls) collected data on a few girls and women. Because there were few sites with sufficient data in any region, we typically reported data for the total number of sites (bolded column percentages in Tables 1, 2, 3, 4 and Tables S1–S4). We report complete data for girls eligible for HPV vaccination, cervical screening, diagnosis, treatment and referral in Tables S6–S11. We qualitatively summarized and reported good practices observed during the site visits. All analyses were performed with Stata 16 SE (Stata Corp., College Station, TX, USA).

**Table 1:** Respondent and site characteristics

Region (no. of sites)	Central Africa (*n* = 7)	East Africa (*n* = 8)	Southern Africa (*n* = 9)	West Africa (*n* = 6)	Total (*n* = 30)
Variables	*N* (%)	*N* (%)	*N* (%)	*N* (%)	*N* (%)
**Respondent's role in the programme**					
Data manager	5 (56)	0 (0)	4 (44)	0 (0)	**9 (30)**
Nurse	0 (0)	2 (100)	0 (0)	0 (0)	**2 (7)**
Physician	2 (22)	3 (33)	2 (22)	2 (22)	**9 (30)**
Programme manager	0 (0)	3 (38)	1 (13)	4 (50)	**8 (27)**
Research manager/assistant	0 (0)	0 (0)	2 (100)	0 (0)	**2 (7)**
**Facility location**					
Urban	7 (28)	7 (28)	5 (20)	6 (24)	**25 (83)**
Rural	0 (0)	1 (20)	4 (80)	0 (0)	**5 (17)**
**Facility type**					
Public	5 (23)	7 (32)	8 (36)	2 (9)	**22 (73)**
NGO	1 (20)	1 (20)	1 (20)	2 (40)	**5 (13)**
FBO	1 (100)	0 (0)	0 (0)	0 (0)	**1 (3)**
Other	0 (0)	0 (0)	0 (0)	2 (100)	**2 (7)**
**Service integration**					
Within ART clinic using existing staff	2 (14)	4 (29)	2 (14)	6 (43)	**14 (47)**
In another unit in hospital where ART clinic is located	4 (30)	3 (23)	6 (46)	0 (0)	**13 (43)**
Off‐site	1 (50)	0 (0)	1 (50)	0 (0)	**2 (7)**
**Screen and treat approach used** ^a^					
Yes	1 (4)	8 (35)	9 (39)	5 (22)	**23 (77)**
No	5 (83)	0 (0)	0 (0)	1 (17)	**6 (20)**
Unknown	1 (100)	0 (0)	0 (0)	0 (0)	**1 (3)**
**Single visit approach used** ^b^					
Yes	2 (10)	5 (25)	7 (35)	6 (30)	**20 (67)**
No	5 (50)	3 (30)	2 (20)	0 (0)	**10 (33)**

*Note*: Total percentages are column percentages in bold, and percentages per region are row percentages.

Abbreviations: ART, antiretroviral therapy; FBO, faith‐based organization; NGO, non‐governmental organization.

^a^ Treatment could be offered during another visit after screening.

^b^ Screening and treatment are offered during the same visit.

**Table 2:** Organization of screening, demand generation and financing

Region (no. of sites)	Central Africa (*n* = 7)	East Africa (*n* = 8)	Southern Africa (*n* = 9)	West Africa (*n* = 6)	Total (*n* = 30)
**Variables**	*N* (%)	*N* (%)	*N* (%)	*N* (%)	***N* (%)**
**Nature of screening programme**					
Pilot	1 (50)	0 (0)	1 (50)	0(0)	**2 (7)**
Routine care	6 (30)	7 (35)	7 (35)	0 (0)	**20 (67)**
Research project	0 (0)	2 (33)	0 (0)	4 (67)	**5 (17)**
**Individual or team for screening coordination**					
Yes	5 (20)	7 (28)	7 (28)	6 (24)	**25 (83)**
No	1 (33)	0 (0)	2 (67)	0 (0)	**3 (10)**
Unknown	1 (100)	0 (0)	0 (0)	0 (0)	**1 (3)**
**Pilot before screening implementation**					
Yes	0 (0)	4 (44)	1 (11)	4 (44)	**9 (30)**
No	5 (36)	3 (21)	4 (29)	2 (14)	**14 (47)**
Unknown	2 (29)	1 (14)	4 (57)	0 (0)	**7 (23)**
**Pilot evaluated**					
Yes, report published	0 (0)	2 (50)	0 (0)	2 (50)	**4 (13)**
Yes, report not published	0 (0)	1 (100)	0 (0)	0 (0)	**1 (3)**
No	0 (0)	0 (0)	0 (0)	1 (100)	**1 (3)**
Unknown	0 (0)	1 (33)	1 (33)	1 (33)	**3 (10)**
**Screening policy available**					
Yes	3 (13)	7 (30)	7 (30)	6 (26)	**23 (77)**
No	1 (33)	0 (0)	2 (67)	0 (0)	**3 (10)**
Unknown	3 (75)	1 (25)	0 (0)	0 (0)	**4 (13)**
**Screening guideline available**					
Yes	2 (10)	7 (33)	6 (29)	6 (29)	**21 (70)**
No	3 (50)	1 (17)	2 (33)	0 (0)	**6 (20)**
Unknown	2 (67)	0 (0)	1 (33)	0 (0)	**3 (10)**
**Initiatives for population awareness by Health Ministry**					
Yes	4 (17)	7 (30)	6 (26)	6 (26)	**23 (77)**
No	2 (67)	0 (0)	1 (33)	0 (0)	**3 (10)**
Unknown	1 (33)	0 (0)	2 (67)	0 (0)	**3 (10)**
**Awareness approach**					
Mass media campaign	1 (5.6)	7 (39)	5 (28)	5 (28)	**18 (78)**
Small media campaign	0 (0)	1 (14)	1 (14)	5 (71)	**7 (30)**
Group education	4 (24)	5 (29)	3 (18)	5 (29)	**17 (74)**
One‐on‐one education	0 (0)	3 (30)	3 (30)	4 (40)	**10 (44)**
Unknown	1 (100)	0 (0)	0 (0)	0 (0)	**1 (3)**
**Invitation system for eligible population**					
Yes	0 (0)	4 (50)	2 (25)	2 (25)	**8 (27)**
No	6 (30)	3 (15)	7 (35)	4 (20)	**20 (67)**
Unknown	1 (100)	0 (0)	0 (0)	0 (0)	**1 (3)**
**Invitation method**					
SMS	0 (0)	0 (0)	1 (50)	1 (50)	**2 (25)**
Phone calls	0 (0)	2 (50)	1 (25)	1 (25)	**4 (50)**
Home visits by health workers	0 (0)	1 (25)	1 (25)	2 (50)	**4 (50)**
Sensitization during consultation	0 (0)	0 (0)	0 (0)	1 (100)	**1 (13)**
Word of mouth	0 (0)	0 (0)	1 (100)	0 (0)	**1 (13)**
Through media (radio, TV), One‐on‐one education	0 (0)	1 (100)	0 (0)	0 (0)	**1 (13)**
**System to invite selected populations**					
Not screened in previous round	0 (0)	5 (71)	1 (14)	1 (14)	**7 (23)**
High‐risk populations only	1 (13)	3 (38)	4 (50)	0 (0)	**8 (27)**
No system	3 (25)	1 (8)	3 (25)	5 (42)	**12 (40)**
Unknown	2 (67)	1 (33)	0 (0)	0 (0)	**3 (10)**
**High‐risk criteria**					
HIV positive	0 (0)	3 (75)	1 (25)	0 (0)	**4 (50)**
HIV positive with menstruation complications	1 (100)	0 (0)	0 (0)	0 (0)	**1 (13)**
Referred from ART clinic	0 (0)	0 (0)	1 (100)	0 (0)	**1 (13)**
Women with high‐risk HPV	0 (0)	0 (0)	1 (100)	0 (0)	**1 (13)**
**Government allocated budget for CC prevention**					
Yes	0 (0)	5 (39)	5 (39)	3 (23)	**13 (43)**
No	5 (39)	2 (15)	3 (28)	3 (23)	**13 (43)**
Unknown	2 (50)	1 (25)	1 (25)	0 (0)	**4 (13)**
**NGO support for health facility**					
Yes	4 (16)	8 (32)	9 (36)	4 (16)	**25 (83)**
No	2 (50)	0 (0)	0 (0)	2 (50)	**4 (13)**
**NGO support for cervical cancer prevention**					
Yes	0 (0)	5 (39)	7 (54)	1 (8)	**13 (43)**
No	7 (41)	3 (18)	2 (12)	5 (29)	**17 (57)**
**Vaccination free of charge (in sites currently offering vaccination or who did in the past)**					
Yes	5 (29)	5 (29)	5 (29)	2 (12)	**17 (100)**
**Diagnosis for pre‐cancer and CC free of charge**					
Yes	0 (0)	3 (38)	4 (50)	1 (13)	**8 (27)**
No	5 (39)	2 (15)	1 (8)	5 (39)	**13 (43)**
Partially	0 (0)	0 (0)	2 (100)	0 (0)	**2 (7)**
Unknown	2 (40)	2 (40)	1 (20)	0 (0)	**5 (17)**
**Treatment for pre‐cancer and cancer treatment free of charge**					
Yes	1 (11)	2 (22)	6 (67)	0 (0)	**9 (30)**
No	4 (40)	0 (0)	1 (10)	5 (50)	**10 (33)**
Partially	0 (0)	3 (50)	2 (33)	1 (17)	**6 (20)**
Unknown	2 (50)	2 (50)	0 (0)	0 (0)	**4 (13)**

*Note*: Total percentages are column percentages in bold, and percentages per region are row percentages.

Abbreviations: ART, anti‐retroviral therapy; CC, cervical cancer; HPV, human papillomavirus.

**Table 3:** Screening, triage and treatment of pre‐cancerous lesions

Region (no. of sites)	Central Africa (*n* = 7)	East Africa (*n* = 8)	Southern Africa (*n* = 9)	West Africa (*n* = 6)	Total (*n* = 30)
**Variables**	*N* (%)	*N* (%)	*N* (%)	*N* (%)	***N* (%)**
**Eligibility**					
All women on ART	2 (17)	3 (25)	5 (42)	2 (17)	**12 (40)**
Other age ranges in years					
15–55	1 (50)	0 (0)	1 (50)	0 (0)	**2 (7)**
18–65	0 (0)	0 (0)	0 (0)	3 (100)	**3 (10)**
30–50	0 (0)	1 (50)	0 (0)	1 (50)	**2 (7)**
>35	1 (100)	0 (0)	0 (0)	0 (0)	**1 (3)**
25–49	0 (0)	0 (0)	1 (0)	0 (0)	**1 (3)**
Sexually active	0 (0)	0 (0)	1 (0)	0 (0)	**1 (3)**
Screening tests used^a^					
Cytology	1 (11)	2 (22)	3 (33)	3 (33)	**9 (30)**
VIA	4 (16)	7 (28)	8 (32)	6 (24)	**25 (83)**
VIAC	0 (0)	1 (13)	6 (75)	1 (13)	**8 (27)**
VILI	1 (20)	1 (20)	0 (0)	3 (60)	**5 (17)**
HPV DNA	0 (0)	3 (25)	6 (50)	3 (25)	**12 (40)**
**Triage test used** ^a^					
Cytology	0 (0)	2 (67)	1 (33)	0 (0)	**3 (10)**
HPV DNA	0 (0)	0 (0)	1 (50)	1 (50)	**2 (7)**
Colposcopy	0 (0)	2 (67)	1 (33)	0 (0)	**3 (10)**
VIA	0 (0)	3 (25)	6 (50)	3 (25)	**12 (40)**
Biopsy	0 (0)	1 (100)	0 (0)	0 (0)	**1 (3)**
None	3 (43)	1 (14)	1 (14)	2 (29)	**7 (23)**
**Testing considerations for post‐menopausal women**					
Yes	1 (9)	4 (36)	2 (18)	4 (36)	**11 (37)**
No	4 (25)	3 (19)	7 (44)	2 (13)	**16 (53)**
Unknown	2 (100)	0 (0)	0 (0)	0 (0)	**2 (7)**
**Tests used for post‐menopausal women among sites with testing considerations**					
Cytology, on‐site	0 (0)	1 (25)	1 (25)	2 (50)	**4 (36)**
Cytology, referred	1 (17)	3 (50)	0 (0)	2 (33)	**6 (55)**
HPV DNA	0 (0)	0 (0)	1 (100)	0 (0)	**1 (9)**
**Diagnosis available on‐site**					
Yes	0 (0)	4 (31)	4 (31)	5 (39)	**13 (43)**
No	7 (44)	3 (19)	5 (31)	1 (6)	**16 (53)**
**Pre‐cancer diagnosis**					
Colposcopy	1 (11)	2 (22)	3 (33)	3 (33)	**9 (30)**
Histopathology	0 (0)	3 (27)	4 (36)	4 (36)	**11 (37)**
Cytology	0 (0)	0 (0)	0 (0)	2 (100)	**2 (7)**
Not available	3 (38)	3 (38)	2 (25)	0 (0)	**8 (27)**
**Pre‐cancer treatment** ^b^					
Cryotherapy	3 (16)	6 (32)	4 (21)	6 (32)	**19 (63)**
CKC	0 (0)	1 (13)	2 (25)	5 (63)	**8 (27)**
Thermocoagulation	0 (0)	3 (23)	6 (46)	4 (31)	**13 (43)**
Simple hysterectomy	3 (27)	2 (18)	1 (9)	5 (46)	**11 (37)**
LEEP	1 (6)	5 (29)	5 (29)	6 (35.3)	**17 (57)**
None	3 (100)	0 (0)	0 (0)	0 (0)	**3 (10)**
**Screening intervals for screen‐negative women**					
6 months	0 (0)	1 (100)	0 (0)	0 (0)	**1 (3)**
12 months	3 (19)	5 (31)	3 (19)	5 (31)	**16 (53)**
24 months	0 (0)	0 (0)	4 (80)	1 (20)	**5 (17)**
36 months	0 (0)	1 (50)	1 (50)	0 (0)	**2 (7)**
Unknown	4 (100)	0 (0)	0 (0)	0 (0)	**4 (13)**
5 yearly (if HPV available)	0 (0)	0 (0)	1 (100)	0 (0)	**1 (3)**
**Re‐screening interval after pre‐cancer treatment**					
6 months	3 (33)	3 (33)	2 (22)	1 (11)	**9 (30)**
12 months	0 (0)	2 (14)	7 (50)	5 (36)	**14 (47)**
Unknown	4 (80)	1 (20)	0 (0)	0 (0)	**5 (17)**

*Note*: Total percentages are column percentages in bold, and percentages per region are row percentages.

Abbreviations: CKC, cold knife conisation; HPV DNA, human papillomavirus/deoxyribonucleic acid; LEEP, loop electrosurgical excision procedure; VIA, visual inspection with acetic acid; VIAC, visual inspection with acetic acid and cervicography; VILI, visual inspection with Lugol's iodine.

^a^ Some sites used more than one screening or triage test.

^b^ More than one treatment method used.

**Table 4:** Surveillance systems and data collection

Region (no. of sites)	Central Africa (*n* = 7)	East Africa (*n* = 8)	Southern Africa (*n* = 9)	West Africa (*n* = 6)	Total (*n* = 30)
Variables	*N* (%)	*N* (%)	*N* (%)	*N* (%)	*N* (%)
**Electronic system for data collection and management**					
Yes	7 (26)	7 (26)	7 (26)	6 (22)	**27 (90)**
No (paper forms)	0 (0)	0 (0)	2 (100)	0 (0)	**2 (7)**
**Level electronic system available**					
National	7 (36.8)	2 (10.5)	5 (26)	5 (26)	**19 (63)**
Sub‐national	0 (0)	2 (67)	1 (33)	0 (0)	**3 (10)**
National and Sub‐national	0 (0)	3 (60)	1 (20)	1 (20)	**5 (17)**
Unknown	0 (0)	0 (0)	1 (33)	2 (67)	**3 (10)**
**Electronic system for data aggregation and reporting available**					
Yes	4 (36)	3 (27)	2 (18)	2 (18)	**11 (37)**
No	2 (13)	3 (20)	6 (40)	4 (27)	**15 (50)**
Unknown	1 (33)	1 (33)	1 (33)	0 (0)	**3 (10)**
**Standardized national indicators for CC monitoring available**					
Yes	3 (18)	5 (29)	5 (29)	4 (24)	**17 (57)**
No	2 (33)	0 (0)	2 (33)	2 (33)	**6 (20)**
Unknown	2 (33)	2 (33)	2 (33)	0 (0)	**6 (20)**
**CC prevention and control data collected**					
Yes	0 (0)	5(33)	6 (40)	4 (27)	**15 (50)**
No	6 (50)	2 (17)	2 17)	2 (17)	**12 (40)**
Unknown	1 (50)	0 (0)	1 (50)	0 (0)	**2 (7)**
**Vaccination data collected in sites with ongoing or past programmes**					
Yes	4 (67)	1 (17)	1 (17)	0 (0)	**6 (55)**
No	0 (0)	1 (20)	4 (80)	0 (0)	**5 (46)**
**Key indicators defined in programme**					
Number vaccinated	3 (43)	3(43)	1 (14)	0 (0)	**7 (70)**
Number screened	3 (14)	6 (29)	8 (38)	4 (19)	**21 (70)**
Number screened positive	3 (14)	6 (29)	8 (38)	4 (19)	**21 (70)**
Number further assessed	0 (0)	3 (38)	5 (63)	0 (0)	**8 (27)**
Number treated	1 (7)	3 (20)	8 (53)	3 (20)	**15 (50)**
**Indicators for CC prevention linked to HIV status available**					
Yes	1 (9)	2 (18)	5 (46)	3 (27)	**11 (37)**
No	4 (36)	1 (9)	3 (27)	3 (27)	**11 (37)**
Unknown	2 (40)	2 (40)	1 (20)	0 (0)	**5 (17)**
**CC prevention and care data available for WLHIV**					
Number screened	0 (0)	2 (20)	5 (50)	3 (30)	**10 (33)**
Number treated for pre‐cancer	0 (0)	0 (0)	4 (67)	2 (33)	**6 (20)**
Number treated for CC	0 (0)	2 (33)	3 (50)	1 (17)	**6 (20)**
**Linkage of CC screening data with PBCR**					
Yes, linked to hospital registry	0 (0)	2 (40)	3 (60)	0 (0)	**5 (17)**
Yes, linked to PBCR	0 (0)	0 (0)	1 (33)	2 (67)	**3 (10)**
PBCR exists but data not linked	0 (0)	1 (33)	1 (33)	1 (33)	**3 (10)**
No cancer registry exists	2 (29)	1 (14)	2 (29)	2 (29)	**7 (23)**
Not collecting CC prevention data	6 (50)	2 (17)	2 17)	2 (17)	**12 (40)**
**Client identification**					
Unique national ID number/code	0 (0)	2 (67)	1 (33)	0 (0)	**3 (10)**
Unique national client health number/code	2 (67)	0 (0)	0 (0)	1 (33)	**3 (10)**
Disease‐specific unique identifiers	2 (29)	2 (29)	0 (0)	3 (43)	**7 (23)**
Facility‐specific client number assigned at the first visit	3 (20)	2 (13)	8 (53)	2 (13)	**15 (50)**
No use of ID numbers or codes	0 (0)	1 (100)	0 (0)	0 (0)	**1 (3)**
**Data collected on cancer stage**					
Yes, systematically	1 (9)	5 (46)	2 (18)	3 (27)	**11 (37)**
No or sporadically	0 (0)	0 (0)	6 (75)	2 (25)	**8 (27)**
Unknown	2 (50)	0 (0)	1 (25)	1 (25)	**4 (13)**
**Do you collect data on survival?**					
Yes	1 (14)	3 (43)	1 (14)	2 (29)	**7 (23)**
No	5 (25)	3 (15)	8 (40)	4 (20)	**20 (67)**
Unknown	1 (50)	1 (50)	0 (0)	(0)0	**2 (7)**

Abbreviations: CC, cervical cancer; PBCR, population‐based cancer registry; WLHIV, women living with HIV.

## 3 RESULTS

### 3.1 Sites and respondent characteristics

**Figure 1:**
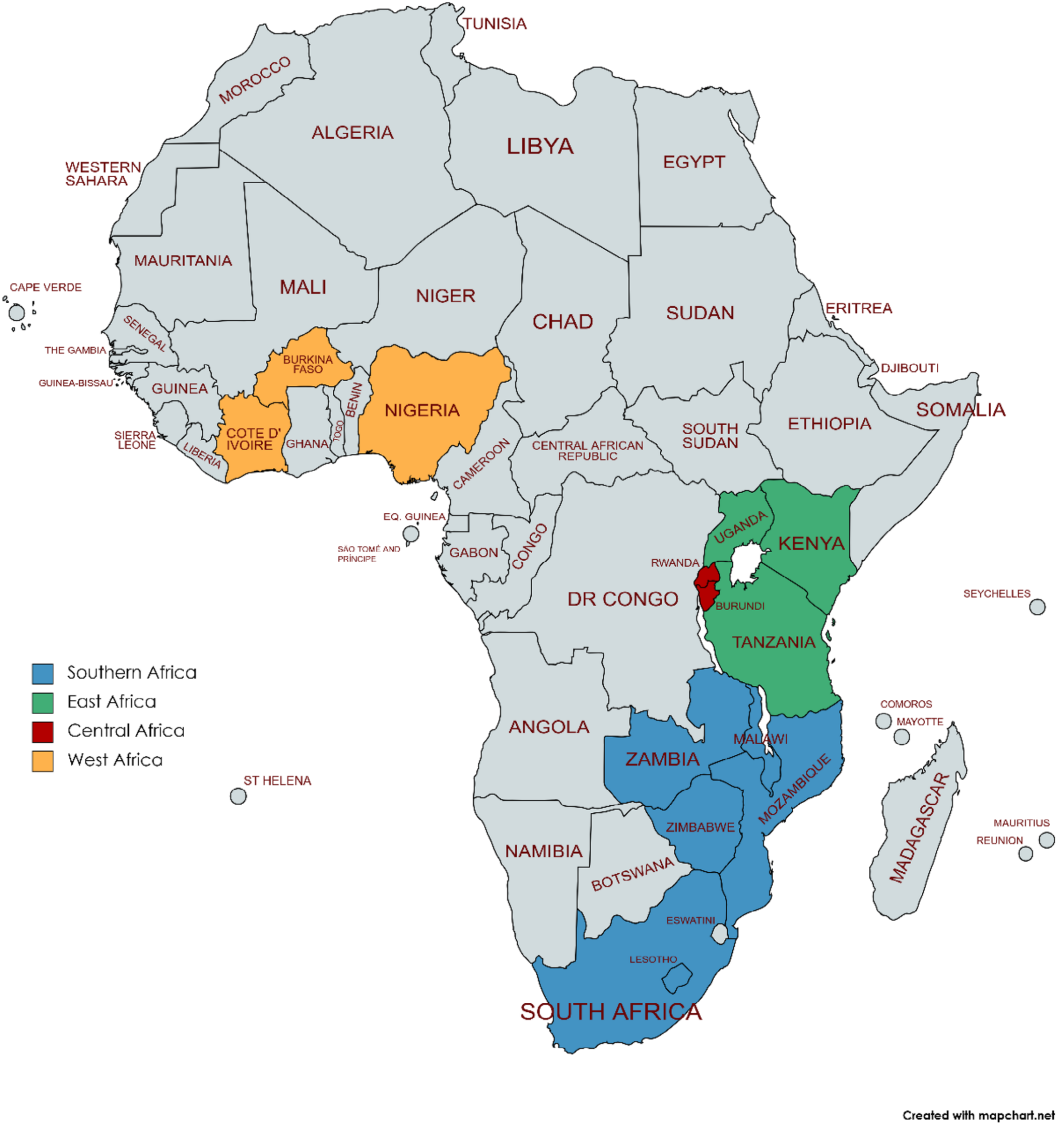
Map showing participating countries.

We included 30 sites across 14 countries in four SSA IeDEA regions: Burundi and Rwanda in central Africa; Kenya, Tanzania and Uganda in East Africa; Lesotho, Malawi, Mozambique, South Africa, Zambia and Zimbabwe in southern Africa; and Burkina Faso, Nigeria and Côte d'Ivoire in West Africa (Figure 1 and Table S12). The survey response rate was 100%. Most respondents were either data managers (30%), physicians (30%) or programme managers (27%). Most sites were public sector facilities (73%) in urban areas (83%; Table 1).

### 3.2 Site‐level data: qualitative indicators

#### 3.2.1 HPV vaccination

Seventeen of 30 sites (57%) had offered (*n* = 7, 23%) or still offered (*n* = 10, 33%) HPV vaccination (Table S1). Vaccination services had been discontinued due to lack of funding (*n* = 3, 43%), vaccination offered once a year (*n* = 2, 29%), low community acceptance and COVID‐19 (*n* = 1, 14%), and completion of pilot/research study (*n* = 1, 14%). HPV vaccines were delivered mostly through a combination of school‐ and community‐based (*n* = 6, 20%) strategies. Of the 10 sites that still provided HPV vaccination, nine sites targeted only girls aged under 15 years. Services were free in all sites that offered HPV vaccination.

#### 3.2.2 Organizing cervical screening, demand generation and programme financing

All included sites offered cervical pre‐cancer screening. These services were often integrated into the HIV clinic and provided by existing staff (47%) or in another unit where the HIV clinic was located, within the larger facility (43%) (Table 1). About a quarter of the CC screening programmes were pilot programmes (*n* = 2, 7%) or research studies (*n* = 5, 17%). Mass media campaigns (78%) and group education (74%) were commonly used to raise demand. Although 83% of sites received financial support from NGOs, less than half of sites (43%) received NGO support specifically designated for CC prevention. Clients paid the total cost (43%) or part of the cost (7%) for diagnosis of suspected cervical pre‐cancer or invasive cancer and the total cost (33%) or part of the cost (20%) for pre‐cancer and cancer treatment (Table 2).

#### 3.2.3 Cervical pre‐cancer screening and pre‐cancer treatment

CC screening was provided on‐site in 93% of facilities, and off‐site in 7%. About an equal number of sites either screened women of any age (40%), or women between 15 and 65 years. The method commonly used to screen (83%) was visual inspection with acetic acid (VIA). HPV DNA testing (40%) and cytology (30%) were performed at less than half of the sites. The most commonly used triage test was VIA (40%). Histopathology (37%) and colposcopy (30%) were commonly used for pre‐cancer diagnosis and usually conducted off‐site (53%). Cryotherapy (63%), thermocoagulation (43%) and loop electrosurgical excision procedure (57%) were the most common pre‐cancer treatment methods. The most common follow‐up interval for screen‐negative women and women treated for pre‐cancer was 12 months (Table 3).

#### 3.2.4 Diagnosis and management of invasive CC

Invasive CC diagnosis (69%) and treatment (67%) services were available in about two‐thirds of the sites (Table S2). Histopathology was the most common diagnostic tool (40%). Simple hysterectomy (37%), radical hysterectomy (53%), chemotherapy (43%) and radiation therapy (40%) were used in combination across sites. Only six (20%) sites reported consistent availability of opioids.

#### 3.2.5 Laboratory testing and quality assurance

Laboratory testing was done either for pre‐cancer only (29%) (HPV DNA testing or cytology), invasive CC diagnosis only (12%) (pathology) or both (59%) (HPV DNA testing, cytology and pathology) (Table S3). Results turnaround time varied between 1 and 4 weeks (65%) in most sites. Quality assurance coordinators who ensured that the screening programmes met quality standards were available in a little over half of the sites (59%); corresponding guidelines were available in 70% of these sites, but in 48% of all sites. Accreditation systems were available in 33% of sites that offered HPV DNA testing and 20% of sites that provided pathology services.

#### 3.2.6 Referral and tracking

Referral for CC screening was most often sporadic (60%); with only 23% consistently referring women for CC screening (Table S4). Of the 25 sites that referred women for pre‐cancer treatment, 40% did so systematically and 43% did so sporadically. Of the 26 sites that referred women for CC treatment, 70% did so systematically. Thirty percent and 47% of sites had no treatment infrastructure for pre‐cancer and CC, respectively. Women who had been referred were usually tracked by phone calls (48%).

#### 3.2.7 Surveillance systems and data collection

The sites mainly relied on electronic data systems (90%) (Table 4); 7 of 10 sites that offered HPV vaccination collected related data, and half the sites collected some data on CC screening. Most sites (70%, *n* = 10) used at least one of the WHO global monitoring indicators for CC elimination, usually the number of girls vaccinated by age 15 years (*n* = 10; 70%), number of women screened (*n* = 30; 70%) and number of women treated (*n* = 30; 50%). Thirty seven percent of sites specifically linked HIV status to existing indicators.

#### 3.2.8 Aggregated data: monitoring indicators reported for girls eligible for HPV vaccination and women in care at HIV clinics

Of the 30 included sites, 11 (37%) collected data for outcome assessment of girls living with HIV and WLHIV, including HPV vaccination, CC screening, pre‐cancer and CC treatment; 37% (*n* = 11) collected some data, but did not disaggregate it by HIV status. Sites receiving financial support from NGOs were more likely to have aggregated patient data informing key performance indicators (73%) as compared to sites that did not have such support (27%) (Table S5).

#### 3.2.9 HPV vaccination

Of the 10 sites that currently offered HPV vaccination, two reported HPV vaccination proportions for 10 or more girls living with HIV and eligible for HPV vaccination at their facility (Table S6); 21% of eligible girls were vaccinated in Newlands Clinic (Zimbabwe), and 88% in Kisesa (Tanzania).

#### 3.2.10 Cervical pre‐cancer screening

**Figure 2:**
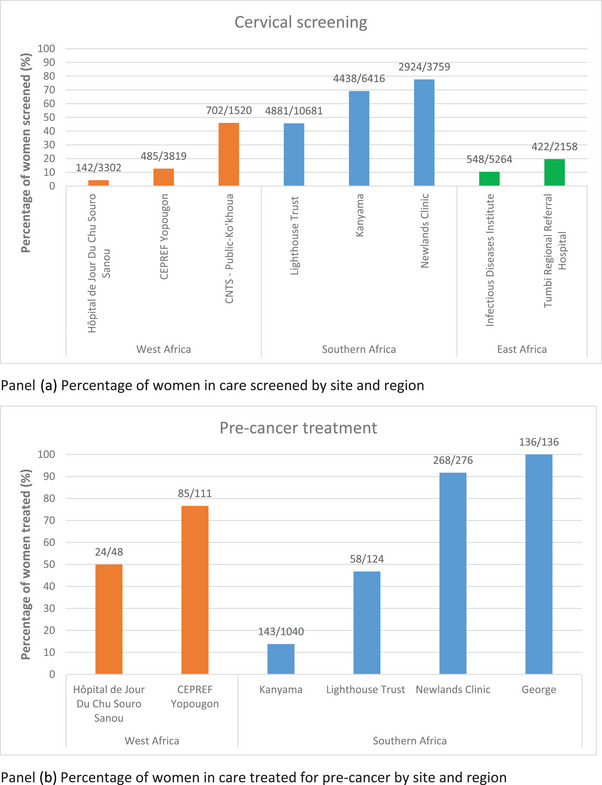
Percentage of women screened for cervical pre‐cancerous lesions (Panel a) and percentage of women treated for cervical pre‐cancer (Panel b). CEPREF, Centre de Prise en charge, de Recherche et de Formation; CNTS, Centre National de Transfusion Sanguine.

Of the 15 sites that reported collecting data on cervical screening, only 11 had disaggregated indicators by HIV status (Table 4). Cervical screening proportions ranged from 4% in Hôpital de Jour du CHU Souro Sanou (Burkina Faso) to 78% in Newlands Clinic (Zimbabwe) (Figure 2, Panel a).

#### 3.2.11 Pre‐cancer treatment and CC management

Pre‐cancer treatment proportions were reported in 10 sites, ranging from 14% in Kanyama Hospital (Zambia) to 100% in George Health Centre (Zambia) (Figure 2, Panel b). Across all sites, there were wide disparities in attrition (proportion of women who did not reach the next necessary step of the cascade) between women whose screens were positive and those who were treated for pre‐cancer, ranging from 0% in George Health Centre to 86% in Kanyama Hospital (Table S8). Only two sites reported data on the number of WLHIV who initiated treatment for CC; three women in Newlands Clinic (Zimbabwe), and one woman in Hôpital de Jour du CHU Souro Sanou (Burkina Faso) (Table S9).

#### 3.2.12 Qualitative summary of good practices

We visited six HIV clinics and two research centres mainly in southern Africa, and recorded some good practices. These included dedicated units and staff for screening, free treatment of precancerous lesions, task shifting for screening and pre‐cancer treatment, capacity enhancement for pathology, unique patient identification, data linkages and partnerships (see Supplement 13 for details).

## 4 DISCUSSION

We surveyed 30 HIV clinics across 14 countries in four SSA IeDEA regions to learn how they implemented CC prevention and care and to populate indicators with routinely collected patient data. Programmes for HPV vaccination were ongoing in only a third of the sites. Less than half of sites always referred women for pre‐cancer and invasive CC diagnosis and treatment, at a fee for women. Almost all sites used electronic systems to collect data, though only half routinely collected CC data, including data needed to inform WHO global monitoring indicators for CC elimination.

WHO recommends HPV vaccination for primary prevention of CC and 41% of WHO member states in the African region had introduced HPV vaccination in their national immunization programmes by the end of 2019 [19]. By the time we conducted our study, some sites ceased vaccinating girls and women against HPV acquisition due to the COVID‐19 pandemic and because of limited financial resources dedicated to HPV vaccination. These findings align with earlier studies that identified barriers to HPV vaccination [20, 21]. GAVI, the Vaccine Alliance, has been trying to address financial barriers for over a decade but funding challenges persist. Although the GAVI model has helped reduce financial barriers, countries must commit to sustaining HPV vaccination programmes as they mature.

The repercussions of the COVID‐19 pandemic are still not clear, but few reports attribute significant interruptions in vaccination programmes to the COVID‐19 pandemic [22, 23]. We found little data on HPV vaccination for girls living with HIV and no previous published studies reported these estimates. Few studies reported data on HPV vaccination rates in the general population and data from countries in SSA are scarce [19, 24]. Since girls living with HIV may receive their booster vaccinations through school‐based programmes, stigma could increase reluctance to get vaccinated and to report. This underserved population may benefit from innovative strategies to deliver vaccines and capture data, and all girls could benefit from programmes that increase preparedness to deliver vaccines during pandemics.

WHO recommends HPV DNA testing and triaging as a cervical screening strategy for WLHIV [3]. Due to the sub‐optimal specificity of the HPV DNA test, triage is essential for WLHIV to distinguish between women who need immediate treatment and those who can be followed up. Although these recommendations were launched towards the end of data collection for our study, a few sites already implemented HPV DNA testing, while maintaining other visual methods for screening and triage. Insufficient infrastructure and financial constraints are obstacles to implementing screen‐triage‐treat strategies at many facilities, and VIA screening remains common [15, 25, 26]. Visual screening is less resource‐intensive and women are likely to be treated the same day they are diagnosed, which increases retention in care [6]. Facilities that wish to transition to HPV DNA testing will have to strengthen their local laboratory infrastructure, improve their quality assurance systems and seek more financing.

Invasive CC management remains challenging in several countries in SSA mostly due to limited infrastructure, limited specialized workforce and unaffordability to women [27]. A recent population‐based cohort study in SSA found that only one in six women with CC received cancer‐directed treatment with curative potential and about two‐thirds of women never accessed treatment [28]. Across sites, women were often referred for invasive CC management and mainly tracked through phone calls/messaging. Since mobile phones proliferate in SSA counties, it is feasible to text women follow‐up reminders [29, 30]. Financial limitations are harder to overcome: earlier studies reported the cost of diagnostic tests, medication and travel as the main financial barriers [31, 32]. At the time of our survey, pre‐cancer and CC diagnosis and treatment services were not free in about two‐thirds of sites. More funding is needed to ensure women's access to invasive CC diagnosis and treatment methods in SSA to improve outcomes as for women in high‐income countries [33].

Routinely collected patient data disaggregated by HIV status were rare at our study sites. Fragmented funding and data systems limit the availability of patient data, making it difficult to improve integrated health programmes [11, 34]. The data available did allow us to see attrition rates varied widely along the steps of CC cascades. For example, attrition rates ranged between 0% (George Health Centre, Zambia) and 86% (Kanyama, Zambia) for women screened positive who should have proceeded to pre‐cancer treatment. A previous study in South Africa reported an attrition rate of about 70% between cascade steps [14], but a similar study conducted in Newlands Clinic (Zimbabwe) found attrition rates were less than 20% between cascade steps [16]. Screening and attrition rates at Newlands may be lower because it receives designated funding for CC prevention and invests in human resources to monitor its programme. Keeping the long‐term benefits of investing in CC prevention in mind, Governments may consider other innovative ways to sustain finance beyond grants. Quality assurance and monitoring are indispensable for any effective CC prevention programme. For monitoring to be feasible, data systems that collect data for pre‐defined indicators in a consistent fashion are crucial. CC prevention facility‐based indicators developed specifically for WLHIV [35] should be considered in these settings. Monitoring CC occurrence and outcomes, including incidence and survival, requires population‐based cancer registries. Where electronic records exist, record linkage of cancer registries and death registries with HIV and CC screening data may help to fill gaps in HIV status and survival data, respectively [6]. Although almost all sites studied had electronic data systems which have been shown to be more efficient in programme monitoring [36], only half of them collected data on CC prevention and care, and less than half linked these data to population and hospital‐based cancer registries. Countries could consider implementing some of the good practices reported in Supplement 13. This could potentially improve efficiency along the screening pathway.

Our study was strengthened by the use of internationally standardized tools to co‐develop our survey with country representatives, improving its validity for each context. Focusing on WLHIV allowed us to identify the needs of this underserved population and see gaps across the CC continuum that may have been overlooked in more general studies. Analysing routinely collected data gave us a clearer picture of the situation on the ground at these sites.

We were also faced with some limitations. Since we included only facilities that belong to the IeDEA consortium receiving some research funding, the situation on the ground may be worse than we describe, especially since we restricted the study to sites with more advanced CC prevention programmes. Also, the service delivery and monitoring landscape for CC may have changed since the time of data collection in some sites.

### 4.1 Policy implications and conclusion

Facility‐based data have contributed significantly to national and global monitoring of HIV. Governments and partners have sought to provide CC prevention and care for WLHIV across SSA and data for monitoring thereof. But insufficient infrastructure and financial challenges hinder these efforts, and impede both monitoring efforts and women's access to HPV vaccination, diagnostic and treatment services as reported across the sites studied. Governments should expand access to treatment infrastructure for cervical pre‐cancer, diagnostic and treatment services for invasive CC, and strengthen linkages between these primary healthcare clinics and referral services. Governments should leverage the existing electronic HIV data systems across these sites to strengthen CC data collection and monitoring. Collecting and analysing these essential data will allow these governments and stakeholders to better plan, target, tailor, and scale‐up sustainable CC prevention and care interventions and track the nation's progress towards the 2030 CC elimination targets in a standardized fashion.

## COMPETING INTERESTS

The authors declared no competing interests.

## AUTHORS’ CONTRIBUTIONS

SLA‐K, MD, K‐GT, AJ, KA, KW‐K, PB, MY, SPB, SB, AM, AS, CC and JB conceived the study, wrote the concept and drafted the survey. TD supported data curation and analysis, BM, CT, GM, HT, JM, OE, OO, MJ and K‐GT coordinated data collection in all sites. SLA‐K and JB wrote the first draft of the manuscript. All co‐authors reviewed and approved the final manuscript.

## FUNDING

This research was funded by the Swiss National Science Foundation (SNSF), under the funding scheme: r4d (Swiss Programme for Research on Global Issues for Development), grant number 177319 (JB, AS). The International Epidemiology Databases to Evaluate AIDS (IeDEA) is supported by the U.S. National Institutes of Health's National Institute of Allergy and Infectious Diseases, the Eunice Kennedy Shriver National Institute of Child Health and Human Development, the National Cancer Institute, the National Institute of Mental Health, the National Institute on Drug Abuse, the National Heart, Lung, and Blood Institute, the National Institute on Alcohol Abuse and Alcoholism, the National Institute of Diabetes and Digestive and Kidney Diseases, and the Fogarty International Center, Central Africa, U01AI096299; East Africa, U01AI069911; Southern Africa, U01AI069924; West Africa, U01AI069919. Informatics resources are supported by the Harmonist project, R24AI24872. This work is solely the responsibility of the authors and does not necessarily represent the official views of any of the institutions mentioned above. SLA‐K also received the Swiss Government Excellence Scholarship, number 2019.0741. Three authors (SLA‐K, MD and TD) received the SSPH+ Global PhD Fellowship Program in Public Health Sciences funded by the European Union's Horizon 2020 research and innovation program under the Marie Skłodowska‐Curie grant agreement No 801076.

## DISCLAIMER

Where authors are identified as personnel of the International Agency for Research on Cancer/World Health Organization, the authors alone are responsible for the views expressed in this article and they do not necessarily represent the decisions, policy or views of the International Agency for Research on Cancer/World Health Organization.

## Supporting information


**Supplement Table 1**: HPV vaccination
**Supplement Table 2**: Cervical cancer diagnosis and treatment/management
**Supplement Table 3**: Laboratory testing and Quality Assurance
**Supplement Table 4**: Referral and tracking
**Supplement Table 5**: Facility characteristics associated with the availability of CC data for WLHIV
**Supplement Table 6**: HPV Vaccination in sites with data for girls living with HIV
**Supplement Table 7**: Cervical screening
**Supplement Table 8**: Treatment of pre‐cancerous lesions: rates according to changing denominators
**Supplement Table 9**: Cervical cancer diagnosis and management
**Supplement Table 10**: Referral for diagnosis and treatment of cervical cancer
**Supplement Table 11**: Number of women screened by type of test
**Supplement Table 12**: List of sites by region and country
**Supplement 13**: Good practices identified in sites visited

## Data Availability

The Supplementary Files contain most of the data that support the findings of our study. Further information is available from the corresponding author upon reasonable request.

## References

[1] World Health Organization . Global strategy to accelerate the elimination of cervical cancer as a public health problem. Geneva: World Health Organization; 2020.

[2] Viviano M , Debeaudrap P , Tebeu P‐M , Tsuala Fouogue J , Vassilakos P , Petignat P . A review of screening strategies for cervical cancer in human immunodeficiency virus‐positive women in sub‐Saharan Africa. Int J Womens Health. 2017;9:69–79. 28203108 10.2147/IJWH.S103868 PMC5298303

[3] WHO guideline for screening and treatment of cervical pre‐cancer lesions for cervical cancer prevention, second edition. Geneva: World Health Organization; 2021. 34314129

[4] World Health Organization . Comprehensive cervical cancer control: a guide to essential practice–2nd ed. 2014. 25642554

[5] World Health Organization . Monitoring national cervical cancer prevention and control programmes: quality control and quality assurance for visual inspection with acetic acid (VIA)‐based programmes. 2013.

[6] UICC/IARC/AFCRN . Cervical cancer elimination in Africa: where we are now and where we need to be? 2022. http://globalink.uicc.org/resources/cervical‐cancer‐elimination‐africa‐where‐are‐we‐now‐and‐where‐do‐we‐need‐be. Accessed 17 June 2022.

[7] Huchko MJ , Maloba M , Nakalembe M , Cohen CR . The time has come to make cervical cancer prevention an essential part of comprehensive sexual and reproductive health services for HIV‐positive women in low‐income countries. J Int AIDS Soc. 2015;18(Suppl 5):20282. 10.7448/IAS.18.6.20282 26643456 PMC4672400

[8] Korn AK , Muzingwani L , O'bryan G , Ensminger A , Boylan AD , Kafidi E‐L , et al. Cervical cancer screening and treatment, HIV infection, and age: program implementation in seven regions of Namibia. PLoS One. 2022;17(2):e0263920. 35171941 10.1371/journal.pone.0263920 PMC8849510

[9] Afzal O , Lieber M , Dottino P , Beddoe AM . Cervical cancer screening in rural South Africa among HIV‐infected migrant farm workers and sex workers. Gynecol Oncol Rep. 2017;20:18–21. 28224134 10.1016/j.gore.2016.12.011 PMC5310162

[10] Sarah Maria N , Olwit C , Kaggwa MM , Nabirye RC , Ngabirano TD . Cervical cancer screening among HIV‐positive women in urban Uganda: a cross sectional study. BMC Womens Health. 2022;22(1):148. 35538482 10.1186/s12905-022-01743-9 PMC9092766

[11] White HL , Meglioli A , Chowdhury R , Nuccio O . Integrating cervical cancer screening and preventive treatment with family planning and HIV‐related services. Int J Gynaecol Obstet. 2017;138(Suppl 1):41–46. 28691337 10.1002/ijgo.12194

[12] Sigfrid L , Murphy G , Haldane V , Chuah FLH , Ong SE , Cervero‐Liceras F , et al. Integrating cervical cancer with HIV healthcare services: a systematic review. PLoS One. 2017;12(7):e0181156. 28732037 10.1371/journal.pone.0181156 PMC5521786

[13] Jansen EEL , Zielonke N , Gini A , Anttila A , Segnan N , Vokó Z , et al. Effect of organised cervical cancer screening on cervical cancer mortality in Europe: a systematic review. Eur J Cancer. 2020;127:207–223. 10.1016/j.ejca.2019.12.013 31980322

[14] Rohner E , Mulongo M , Pasipamire T , Oberlin AM , Goeieman B , Williams S , et al. Mapping the cervical cancer screening cascade among women living with HIV in Johannesburg, South Africa^a^ . Int J Gynaecol Obstet. 2021;152(1):53–59. 33188707 10.1002/ijgo.13485

[15] Pry JM , Manasyan A , Kapambwe S , Taghavi K , Duran‐Frigola M , Mwanahamuntu M , et al. Cervical cancer screening outcomes in Zambia, 2010–19: a cohort study. Lancet Glob Health. 2021;9(6):e832–e840. 34019837 10.1016/S2214-109X(21)00062-0

[16] Taghavi K , Mandiriri A , Shamu T , Rohner E , Bütikofer L , Asangbeh S , et al. Cervical Cancer Screening Cascade for women living with HIV: a cohort study from Zimbabwe. PLOS Glob Public Health. 2022;2(2):e0000156. 36860760 10.1371/journal.pgph.0000156 PMC9974171

[17] World Health Organization . Improving data for decision‐making: a toolkit for cervical cancer prevention and control programmes. Geneva: World Health Organization; 2018.

[18] World Health Organization . HIV strategic information for impact: cascade data use manual: to identify gaps in HIV and health services for programme improvement: user manual. World Health Organization; 2018.

[19] Bruni L , Saura‐Lázaro A , Montoliu A , Brotons M , Alemany L , Diallo MS , et al. HPV vaccination introduction worldwide and WHO and UNICEF estimates of national HPV immunization coverage 2010–2019. Prev Med. 2021;144:106399. 33388322 10.1016/j.ypmed.2020.106399

[20] Amponsah‐Dacosta E , Kagina BM , Olivier J . Health systems constraints and facilitators of human papillomavirus immunization programmes in sub‐Saharan Africa: a systematic review. Health Policy Plan. 2020;35(6):701–717. 32538437 10.1093/heapol/czaa017 PMC7294244

[21] Zheng L , Wu J , Zheng M . Barriers to and facilitators of human papillomavirus vaccination among people aged 9 to 26 years: a systematic review. Sex Transm Dis. 2021;48(12):e255–e262. 33783412 10.1097/OLQ.0000000000001407 PMC8594509

[22] Toor J , Li X , Jit M , Trotter CL , Echeverria‐Londono S , Hartner A‐M , et al. COVID‐19 impact on routine immunisations for vaccine‐preventable diseases: projecting the effect of different routes to recovery. Vaccine. 2022;40(31):4142–4149. 35672179 10.1016/j.vaccine.2022.05.074 PMC9148934

[23] Shet A , Carr K , Danovaro‐Holliday MC , Sodha SV , Prosperi C , Wunderlich J , et al. Impact of the SARS‐CoV‐2 pandemic on routine immunisation services: evidence of disruption and recovery from 170 countries and territories. Lancet Glob Health. 2022;10(2):e186–e194. 34951973 10.1016/S2214-109X(21)00512-X PMC8691849

[24] Spayne J , Hesketh T . Estimate of global human papillomavirus vaccination coverage: analysis of country‐level indicators. BMJ Open. 2021;11(9):e052016. Published 2021 Sep 2. 10.1136/bmjopen-2021-052016 PMC8413939 34475188

[25] Taghavi K , Mandiriri A , Shamu T , Rohner E , Bütikofer L , Asangbeh S , et al. Cervical Cancer Screening Cascade for women living with HIV: a cohort study from Zimbabwe. PLOS Glob Public Health. 2022;2(2):e0000156. 36860760 10.1371/journal.pgph.0000156 PMC9974171

[26] Lee H , Kang Y , Ju W . Cervical cancer screening in developing countries: using visual inspection methods. Clin J Oncol Nurs. 2016;20(1):79–83. 26800410 10.1188/16.CJON.79-83

[27] George Bush Institute . Strategies for accelerating access to treatment for advanced cervical cancer in sub‐Saharan Africa. 2021. www.gofurther.org. Accessed 5 Sept 2023.

[28] Griesel M , Seraphin TP , Mezger NCS , Hämmerl L , Feuchtner J , Joko‐Fru WY , et al. Cervical cancer in sub‐Saharan Africa: a multinational population‐based cohort study of care and guideline adherence. Oncologist. 2021;26(5):e807–e816. 33565668 10.1002/onco.13718 PMC8100544

[29] Erwin E , Aronson KJ , Day A , Ginsburg O , Macheku G , Feksi A , et al. SMS behaviour change communication and eVoucher interventions to increase uptake of cervical cancer screening in the Kilimanjaro and Arusha regions of Tanzania: a randomised, double‐blind, controlled trial of effectiveness. BMJ Innov. 2019;5(1):28–34. 10.1136/bmjinnov-2018-000276 PMC6792319 31645991

[30] Wanyoro AK , Kabiru EW . Use of mobile phone short text message service to enhance cervical cancer screening at Thika Level 5 Hospital, Kiambu County, Kenya: a randomised controlled trial. J Obstet Gynaecol Res. 2017;5(1):10–20. 10.5923/j.rog.20170501.03

[31] Adedimeji A , Ajeh R , Pierz A , Nkeng R , Ndenkeh J , Fuhngwa N , et al. Challenges and opportunities associated with cervical cancer screening programs in a low income, high HIV prevalence context. BMC Womens Health. 2021;21(1):74. 33602194 10.1186/s12905-021-01211-w PMC7890622

[32] Owenga JA , Nyambedha EO . Perception of cervical cancer patients on their financial challenges in Western Kenya. BMC Health Serv Res. 2018;18(1):261. 29631577 10.1186/s12913-018-3073-2 PMC5891984

[33] Marth C , Landoni F , Mahner S , Mccormack M , Gonzalez‐Martin A , Colombo N . Cervical cancer: ESMO Clinical Practice Guidelines for diagnosis, treatment and follow‐up. Ann Oncol. 2017;28(suppl_4):iv72–iv83. 28881916 10.1093/annonc/mdx220

[34] Drummond JL , Were MC , Arrossi S , Wools‐Kaloustian K . Cervical cancer data and data systems in limited‐resource settings: challenges and opportunities. Int J Gynaecol Obstet. 2017;138(Suppl 1):33–40. 28691330 10.1002/ijgo.12192

[35] Davidović M , Asangbeh SL , Taghavi K , Dhokothera T , Jaquet A , Musick B , et al. Facility‐based indicators to manage and scale up cervical cancer prevention and care services for women living with HIV in sub‐Saharan Africa: three‐round modified Delphi consensus method. J Acquir Immune Defic Syndr 2022;95(2):170–178. 10.1097/QAI.0000000000003343 PMC10794028 38211958

[36] Walther B , Hossin S , Townend J , Abernethy N , Parker D , Jeffries D . Comparison of electronic data capture (EDC) with the standard data capture method for clinical trial data. PLoS One. 2011;6(9):e25348. 10.1371/journal.pone.0025348 21966505 PMC3179496

