# Supplementary material for: Cervical cancer prevention and care in HIV clinics across sub‐Saharan Africa: results of a facility‐based survey
Source: J Int AIDS Soc. 2024 Jul 9;27(7):e26303. doi: 10.1002/jia2.26303 (PMC11232047; doi:10.1002/jia2.26303)
Supplement: Supplementary file 1 — Supplement Table 1: HPV vaccination Supplement Table 2: Cervical cancer diagnosis and treatment/management Supplement Table 3: Laboratory testing and Quality Assurance Supplement Table 4: Referral and tracking Supplement Table 5: Facility characteristics associated with the availability of CC data for WLHIV Supplement Table 6: HPV Vaccination in sites with data for girls living with HIV Supplement Table 7: Cervical screening Supplement Table 8: Treatment of pre‐cancerous lesions: rates according to changing denominators Supplement Table 9: Cervical cancer diagnosis and management Supplement Table 10: Referral for diagnosis and treatment of cervical cancer Supplement Table 11: Number of women screened by type of test Supplement Table 12: List of sites by region and country Supplement 13: Good practices identified in sites visited [file JIA2-27-e26303-s001.docx]

**Supplementary Files**

**Supplement Table 1: HPV vaccination**

| **Region (number of sites)** | Central Africa (n=7) | East Africa (n=8) | Southern  Africa (n=9) | West Africa (n=6) | **Total (N=30)** |
| --- | --- | --- | --- | --- | --- |
| **Variables** | N (%) | N (%) | N (%) | N (%) | **N (%)** |
| **HPV vaccination** | | | | | |
| Yes, ongoing | 5 (50) | 3 (30) | 2 (20) | 0 (0) | **10 (33)** |
| Yes, in the past | 0 (0) | 2 (29) | 3 (43) | 2 (29) | **7 (23)** |
| No | 2 (15) | 3 (23) | 4 (31) | 4 (31) | **13 (43)** |
| **Reason vaccination was stopped** | **n=0** | **n=2** | **n=3** | **n=1** | **N=7** |
| Lack of funding | 0 (0) | 1 (33) | 1 (33) | 1 (33) | **3 (43)** |
| Vaccination is given once a year | 0 (0) | 0 (0) | 2 (100) | 0 (0) | **2 (29)** |
| COVID-19 and low community acceptance | 0 (0) | 1 (100) | 0 (0) | 0 (0) | **1 (14)** |
| Research project | 0 (0) | 0 (0) | 0 (0) | 1 (100) | **1 (14)** |
| **HPV vaccination in sites with ongoing or past programs** | **n=5** | **n=5** | **n=5** | **n=2** | **N=17** |
| **Vaccination strategy** | | | | | |
| School-based only | 0 (0) | 2 (67) | 1 (33) | 0 (0) | **3 (10)** |
| School and Community-based | 4 (67) | 0 (0) | 2 (33) | 0 (0) | **6 (20)** |
| School-based, Community based and Campaigns | 1 (100) | 0 (0) | 0 (0) | 0 (0) | **1 (3)** |
| Campaigns only | 0 (0) | 0 (0) | 0 (0) | 1 (100) | **1 (3)** |
| Routine | 0 (0) | 3 (75) | 1 (25) | 0 (0) | **4 (13)** |
| Not applicable/missing | 2 (13) | 3 (20) | 4 (27) | 6 (40) | **15 (50)** |
| **HPV vaccine type** | | | | | |
| Bivalent | 0 (0) | 0 (0) | 2 (50) | 2 (50) | **4 (24)** |
| Quadrivalent | 5 (50) | 4 (40) | 1 (10) | 0 (0) | **10 (59)** |
| Nonavalent | 0 (0) | 0 (0) | 1 (100) | 0 (0) | **1 (6)** |
| Unknown | 0 (0) | 1 (50) | 1 (50) | 0 (0) | **2 (12)** |
| **Target population** | | | | | |
| Girls only | 5 (31) | 5 (31) | 4 (25) | 2 (13) | **16 (94)** |
| Girls and boys | 0 (0) | 0 (0) | 1 (100) | 0 (0) | **1 (6)** |
| **Target age** | | | | | |
| < 15 years old | 5 (31) | 5 (31) | 4 (25) | 2 (13) | **16 (94)** |
| 8-18 years old | 0 (0) | 0 (0) | 1 (11) | 0 (0) | **1 (6)** |
| **HPV vaccination free of charge** | | | | | |
| Yes | 5 (29) | 5 (29) | 5 (29) | 2 (12) | **17 (100)** |

Abbreviation: HPV, Human Papillomavirus. Total percentages are column percentages in bold, and percentages per region are row percentages.

**Supplement Table 2: Cervical cancer diagnosis and treatment/management**

| **Region (number of sites)** | Central Africa | East Africa | Southern Africa­­­­­­­­­­­­­­­­­­­­­­­­­­­­­ | West Africa | **Total** |
| --- | --- | --- | --- | --- | --- |
| **Variables** | n=7 (%) | n=8 (%) | n=9 (%) | n=6 (%) | **N=30 (%)** |
| **Cancer diagnosis** | | | | | |
| Histopathology | 1 (8) | 3 (25) | 4 (33) | 4 (33) | **12 (40)** |
| Biopsy sent to South Africa | 0 (0) | 0 (0) | 1 (100) | 0 (0) | **1 (3)** |
| Referred | 0 (0) | 2 (100) | 0 (0) | 0 (0) | **2 (7)** |
| Clinical | 0 (0) | 0 (0) | 0 (0) | 2 (100) | **2 (7)** |
| Tomodensitometry | 0 (0) | 0 (0) | 0 (0) | 1 (100) | **1 (3)** |
| Not available | 3 (38) | 2 (25) | 3 (38) | 0 (0) | **8 (27)** |
| **Cancer treatment** | | | | | |
| Simple hysterectomy | 1 (9) | 4 (36) | 2 (18) | 4 (36) | **11 (37)** |
| Radical hysterectomy | 2 (13) | 5 (31) | 3 (19) | 6 (38) | **16 (53)** |
| Chemotherapy | 1 (8) | 4 (31) | 3 (23) | 5 (39) | **13 (43)** |
| Radiation therapy | 0 (0) | 5 (42) | 2 (17) | 5 (42) | **12 (40)** |
| Intra-cavitary radiation | 0 (0) | 0 (0) | 2 (50) | 2 (50) | **4 (13)** |
| None | 3 (30) | 2 (20) | 5 (50) | 0 (0) | **10 (33)** |
| **Access to opioids** | | | | | |
| Always | 0 (0) | 1 (17) | 3 (50) | 2 (33) | **6 (20)** |
| Sometimes | 1 (33) | 2 (67) | 0 (0) | 0 (0) | **3 (10)** |
| Never | 6 (30) | 4 (20) | 6 (30) | 4 (20) | **20 (67)** |

Total percentages are column percentages in bold, and percentages per region are row percentages.

**Supplement Table 3: Laboratory testing and Quality Assurance**

| **Region (number of sites)** | Central Africa | East Africa | Southern Africa­­­­­­­­­­­­­­­­­­­­­­­­­­­­­ | West Africa | **Total** |
| --- | --- | --- | --- | --- | --- |
| **Variables** | n=7 (%) | n=8 (%) | n=9 (%) | n=6 (%) | **N=30 (%)** |
| **Laboratory testing** |  |  |  |  |  |
| **Laboratory testing for pre-cancer only** | | | | | |
| Yes | 0 (0) | 1 (20) | 2 (40) | 2 (40) | **5 (29)** |
| **Laboratory testing (diagnosis) for invasive cancer only** | | | | | |
| Yes | 0 (0) | 0 (0) | 2 (100) | 0 (0) | **2 (12)** |
| **Laboratory testing (diagnosis) for both pre-cancer and invasive cancer** | | | | | |
| Yes | 0 (0) | 4 (40) | 3 (30) | 3 (30) | **10 (59)** |
| **Time between sample collection and arrival at laboratory** | | | | | |
| 1 day | 0 (0) | 2 (29) | 2 (29) | 3 (43) | **7 (41)** |
| 2-7 days | 0 (0) | 2 (29) | 5 (71) | 0 (0) | **7 (41)** |
| Same day HPV, 2 weeks for cytology and histopathology | 0 (0) | 0 (0) | 0 (0) | 1 (100) | **1 (6)** |
| No specific time | 0 (0) | 1 (100) | 0 (0) | 0 (0) | **1 (6)** |
| Sample collection in laboratory | 0 (0) | 0 (0) | 0 (0) | 1 (100) | **1 (6)** |
| **Results turn- around time** | | | | | |
| Same day for HPV/ 2 months for cytology and histology | 0 (0) | 0 (0) | 0 (0) | 1 (100) | **1 (7)** |
| <1 week | 0 (0) | 1 (50) | 1 (50) | 0 (0) | **2 (13)** |
| 1-4 weeks | 0 (0) | 3 (27) | 5 (45) | 3 (27) | **11 (65)** |
| 5-6 weeks | 0 (0) | 1 (33) | 1 (33) | 1 (33) | **3 (20)** |
| **Results reception format** | | | | | |
| Electronic | 0 (0) | 2 (29) | 3 (43) | 2 (29) | **7 (23)** |
| Paper format | 2 (11) | 4 (22) | 7 (39) | 5 (28) | **18 (60)** |
| **Transfer of results** | | | | | |
| Results are sent to clinic | 2 (14) | 3 (21) | 6 (43) | 3 (21) | **14 (47)** |
| Staff actively go search for them | 0 (0) | 2 (33) | 2 (33) | 2 (33) | **6 (20)** |
| **Time between results reception and communication to client** | | | | | |
| Within 7 days | 0 (0) | 5 (42) | 4 (33) | 3 (25) | **12 (40)** |
| Women asked to return in 2 weeks | 0 (0) | 0 (0) | 1 (100) | 0 (0) | **1 (3)** |
| About 30 days (during next HIV appointment) | 0 (0) | 0 (0) | 2 (100) | 0 (0) | **2 (7)** |
| **Quality Assurance** | | | | | |
| **Quality assurance policy or guideline available** | | | | | |
| Yes | 2 (14) | 6 (43) | 3 (21) | 3 (21) | **14 (48)** |
| No | 3 (33) | 0 (0) | 3 (33) | 3 (33) | **9 (30)** |
| Unknown | 2 (33) | 1 (17) | 3 (50) | (0)0 | **6 (21)** |
| **Quality assurance coordinator or team available** | | | | | |
| Yes | 1 (6) | 7 (41) | 6 (35.3) | 3 (18) | **17 (59)** |
| No | 4 (44) | (0) 0 | 2 (22) | 3 (33) | **9 (31)** |
| Unknown | 2 (67) | (0) 0 | 1 (33) | (0)0 | **3 (10)** |
| **System of accreditation for HPV** | | | | | |
| Yes | 0(0)* | 5 (50) | 5 (50) | (0)0 | **10 (33)** |
| No | 5 (31) | 2 (13) | 3 (19) | 6 (38) | **16 (53)** |
| Unknown | 1 (50) | (00 | 1 (50) | (0)0 | **2 (7)** |
| **System of accreditation for pathology** | | | | | |
| Yes | 1 (17) | 1 (17) | 3 (50) | 1 (17) | **6 (20)** |
| No | 4 (27) | 3 (20) | 4 (27) | 4 (27) | **15 (50)** |
| Unknown | 2 (25) | 3 (38) | 2 (25) | 1 (13) | **8 (27)** |

Abbreviation: HPV, Human Papillomavirus. Total percentages are column percentages in bold, and percentages per region are row percentages.

**Supplement Table 4: Referral and tracking**

| **Region (number of sites)** | Central Africa | East Africa | Southern Africa­­­­­­­­­­­­­­­­­­­­­­­­­­­­­ | West Africa | **Total** |
| --- | --- | --- | --- | --- | --- |
| **Variables** | n=7 (%) | n=8 (%) | n=9 (%) | n=6 (%) | **N=30 (%)** |
| **SCREENING** | | | | | |
| **Referral for screening** | | | | | |
| Always | 5 (71) | 1 (14) | 1 (14) | 0 (0) | **7 (23)** |
| Sometimes | 2 (11) | 4 (22) | 6 (33) | 6 (33) | **18 (60)** |
| Never | 0 (0) | 2 (67) | 1 (33) | 0 (0) | **3 (10)** |
| Missing | 0 (0) | 1 (3) | 1 (3) | 0 (0) | **2 (7)** |
| **Receiving site for referral** |  |  |  |  |  |
| On-site | 3 (30) | 3 (30) | 3 (30) | 1 (10) | **10 (33)** |
| Off-site | 4 (31) | 2 (15) | 3 (23) | 4 (31) | **13 (43)** |
| Missing | 0 (0) | 0(0) | 1 (50) | 1(50) | **2 (7)** |
| Not applicable | 0 (0) | 3 (60) | 2 (40) | 0 (0) | **5 (17)** |
| **Reason for screening referral*** |  |  |  |  |  |
| Screening services available in another unit in hospital | 3 (33) | 2 (22) | 3 (33) | 1 (11) | **9 (38)** |
| Screening services available but not always functional | 0 (0) | 0 (0) | 0 (0) | 1 (100) | **1 (4)** |
| Screening services not available on-site | 2 (50) | 0 (0) | 1 (25) | 1 (25) | **4 (17)** |
| For diagnosis (suspect cancer) | 2 (20) | 3 (30) | 2 (20) | 3 (30) | **10 (42)** |
| Missing | 0 (0) | 0 (0) | 1 (100) | 0 (0) | **1(3)** |
| **Do you keep track of women referred for screening?** | | | | | |
| Always | 3 (17) | 5 (28) | 6 (33) | 4 (22) | **18 (60)** |
| Sometimes | 2 (40) | 0 (0) | 1 (20) | 2 (40) | **5 (17)** |
| Never | 2 (33) | 2 (33) | 2 (33) | 0 (0) | **6 (20)** |
| Missing | 0 (0) | 1 (100) | 0 (0) | 0 (0) | **1 (3)** |
| **Tracing strategy** | | | | | |
| Phone call | 1 (8) | 3 (25) | 4 (33) | 4 (33) | **12 (48)** |
| Trace from HIV clinic | 3 (30) | 2 (20) | 3 (30) | 2 (20) | **10 (33)** |
| Missing | 3 (100) | 0 (0) | 0 (0) | 0 (0) | **3 (10)** |
| Not applicable | 0 (0) | 3 (60) | 2 (40) | 0 (0) | **5 (17)** |
| **Trace lab results not received** | | | | | |
| Always | 0 (0) | 5 (31) | 6 (38) | 5 (31) | **16 (94)** |
| Sometimes | 0 (0) | 0 (0) | 1 (100) | 0 (0) | **1 (6)** |
| Not applicable | 7 (54) | 3 (23.0) | 2 (15) | 1 (8) | **13 (43)** |
| **Tracing strategy** | | | | | |
| Send a query to the lab | 0 (0) | 4 (31) | 6 (46) | 3 (23) | **13 (77)** |
| Re-invite women for screening | 0 (0) | 0 (0) | 1 (50) | 1 (50) | **2 (12)** |
| Visit the laboratory | 0 (0) | 0 (0) | 0 (0) | 1 (100) | **1 (6)** |
| Repeat Pap smear | 0 (0) | 0 (0) | 1 (100) | 0 (0) | **1 (6)** |
| Not applicable | 7 (54) | 3 (23) | 2 (15) | 1 (8) | **13 (43)** |
| **PRE-CANCER** | | | | | |
| **Do you contact women for pre-cancer treatment?** | | | | | |
| Always | 2 (12) | 5 (29) | 4 (24) | 6 (35.3) | **17 (57)** |
| Sometimes | 1 (20) | 1 (20) | 3 (60) | 0 (0) | **5 (17)** |
| Never | 3 (50) | 1 (17) | 2 (33) | 0 (0) | **6 (20)** |
| Missing | 1 (50) | 1 (50) | 0 (0) | 0 (0) | **2 (7)** |
| **Do you refer women for pre-cancer treatment?** | | | | | |
| Always | 4 (33) | 3 (25) | 1 (8) | 4 (33) | **12 (40)** |
| Sometimes | 1 (8) | 3 (23) | 7 (54) | 2 (15) | **13 (43)** |
| Never | 2 (50) | 1 (25) | 1 (25) | 0 (0) | **4 (13)** |
| Missing | 0 (0) | 1 (100) | 0 (0) | 0 (0) | **1 (3)** |
| **Reasons for pre-cancer treatment referral** | | | | | |
| No treatment infrastructure | 3 (33) | 2 (22) | 1 (11) | 3 (33) | **9 (30)** |
| Large lesion/suspect cancer | 1 (8) | 3 (25) | 5 (42) | 3 (25) | **12 (40)** |
| Need for specialised care | 0 (0) | 1 (50) | 1 (50) | 0 (0) | **2 (7)** |
| Missing | 1 | 0 (0) | 1 | 0 (0) | **2 (7)** |
| Not applicable | 2 | 1 | 1 | 0 (0) | **5 (17)** |
| **Contact for follow-up after pre-cancer treatment** | | | | | |
| Always | 1 (7) | 5 (33) | 4 (27) | 5 (33) | **15 (50)** |
| Sometimes | 2 (29) | 2 (29) | 3 (43) | 0 (0) | **7 (23)** |
| Never | 4 (57) | 0 (0) | 2 (29) | 1 (14) | **7 (23)** |
| Missing | 0 (0) | 1 (100) | 0 (0) | 0 (0) | **1 (3)** |
| **CERVICAL CANCER** | | | | | |
| **Do you contact women for cancer treatment?** | | | | | |
| Always | 2 (14) | 5 (36) | 3 (21) | 4 (29) | **14 (47)** |
| Sometimes | 2 (33) | 1 (17) | 1 (17) | 2 (33) | **6 (20)** |
| Never | 2 (25) | 1 (13) | 5 (63) | 0 (0) | **8 (27)** |
| Missing | 1 (50) | 1 (50) | 0 (0) | 0 (0) | **2 (7)** |
| **Referral for cancer treatment** | | | | | |
| Always | 3 (14) | 5 (24) | 7 (33) | 6 (29) | **21 (70)** |
| Sometimes | 1 (20) | 2 (40) | 2 (40) | 0 (0) | **5 (17)** |
| Never | 3 (43) | 0 (0) | 0 (0) | 0 (0) | **3 (10)** |
| Missing / unknown | 0 (0) | 1 (100) | 0 (0) | 0 (0) | **1 (3)** |
| **Reasons for cancer treatment referral** | | | | | |
| No treatment infrastructure | 3 (21) | 3 (21) | 5 (36) | 3 (21) | **14 (47)** |
| Need for specialised care | 0 (0) | 4 (40) | 3 (30) | 3 (30) | **10 (33)** |
| Missing | 1 (50) | 0 (0) | 1 (50) | 0 (0) | **2 (7)** |
| Not applicable | 3 (75) | 1 (25) | 0 (0) | 0 (0) | **4 (13)** |
| **Contact for follow-up after cancer treatment** | | | | | |
| Always | 1 (9) | 5 (46) | 1 (9) | 4 (36) | **11 (37)** |
| Sometimes | 1 (20) | 1 (20) | 3 (60) | 0 (0) | **5 (17)** |
| Never | 5 (39) | 1 (8) | 5 (39) | 2 (15) | **13 (43)** |
| Missing | 0 (0) | 1 (100) | 0 (0) | 0 (0) | **1 (3)** |

Total percentages are column percentages in bold, and percentages per region are row percentages.

Supplement table 5: Facility characteristics associated with the availability of CC data for WLHIV

| **CC data for WLHIV available** | **Facility characteristic** | | | **p-value** | |
| --- | --- | --- | --- | --- | --- |
|  | **Facility location** | | |  | |
|  | Rural | Urban | - |  | |
| Yes | 2 (18) | 9 (82) | - | 1.00 | |
| No | 1 (9) | 10 (91) | - |  | |
| Missing | 2 (25) | 6 (75) | - |  | |
|  | **Facility type** | | |  |  |
|  | Public | NGO | Other |  | |
| Yes | 8 (73) | 3 (27) | 0 (0) | 0.32 | |
| No | 8 (73) | 1 (9) | 2 (18) |  | |
| Missing | 6 (75) | 1 (13) | 1 (13) |  | |
|  | **Services integration** | | |  | |
|  | In another unit within HIV clinic premises | Within HIV clinic | Off-site |  | |
| Yes | 3 (27) | 8 (73) | 0 (0) | 0.12 | |
| No | 6 (55) | 3 (27) | 2 (18) |  | |
| Missing | 4 (50) | 4 (50) | 0 (0) |  | |
|  | **NGO support for CC prevention** | | |  | |
|  | Yes | No |  |  | |
| Yes | 8 (73) | 3 (27) | - | **0.03** | |
| No | 2 (17) | 9 (82) | - |  | |
| Missing | 3 (38) | 5 (63) | - |  | |

**Supplement Table 6: HPV Vaccination in sites with data for girls living with HIV**

| **Region** | **Country Name** | **Facility Name** | **Index year** | **Eligibility criteria (age in years)** | **# of eligible girls** | **# of eligible young women (15-26 years)** | **Vaccinated against HPV before 15 years old** | **Vaccinated against HPV after 15 years old** |
| --- | --- | --- | --- | --- | --- | --- | --- | --- |
| **Central Africa** | Rwanda | Gikondo HC | 2018 | 10-14 | 4 | 4 | 4 (100) | ~~-~~ |
|  | Rwanda | Masaka HC | 2018 | 12 | 2 | 62 | 2 (100) | 2 (3) |
| **East Africa** | Tanzania | Kisesa HC | - | - | 25 | 32 | 22 (88) | 29 (91) |
| **Southern Africa** | Zimbabwe | Newlands Clinic | 2019 | 8-18 | 24 | 3 | 5 (21) | 2 (67) |

Abbreviations: HC, Health Centre; #, number

**Supplement Table 7: Cervical screening**

| **Region and Facility Name** | **Index year** | **Women in care** | **Screened** | | **Screen negative** | | **Screen positive** | |
| --- | --- | --- | --- | --- | --- | --- | --- | --- |
|  | Calendar year | **n** | **n** | Rate [%]^a^ | **n** | Rate [%]^b^ | **n** | Rate [%]^c^ |
| Year screening programme started † | | | | | | | | |
| **Data for WLHIV** | | | | | | | | |
| **Central Africa** |  |  |  |  |  |  |  |  |
| Kabuga HC | - | - | - |  | - |  | - |  |
| **East Africa** |  |  |  |  |  |  |  |  |
| Tumbi Regional Referral Hospital | 2020 | 2158 | 422 | 20 | - |  | 5 | 1.2 |
| Infectious Diseases Institute | 2015 | 5264 | 548 | 10 | 484 | 88 | 60 | 11 |
| **Southern Africa** |  |  |  |  |  |  |  |  |
| Seboche Mission Hospital | - | - | - | - | - |  | - |  |
| Lighthouse Trust | 2020 | 10681 | 4881 | 46 | 4234 | 87 | 124 | 3 |
| Kanyama | 2020 | 6416 | 4438 | 69 | 3343 | 75 | 1040 | 23 |
| George HC | 2020 | 419 | 3731 | g | 3575 | 96 | 136 | 4 |
| Newlands Clinic | 2019 | 3759 | 2924 | 78 | 2624 | 90 | 276 | 9 |
| **West Africa** |  |  |  |  |  |  |  |  |
| CEPREF Yopougon | 2017 | 3819 | 485 | 1 | 371 | 77 | 111 | 2 |
| CNTS - Public-Ko'khoua | 2019 | 1520 | 702 | 46 | 674 | 96 | 28 | 4 |
| Hôpital de Jour Du Chu Souro Sanou | 2019 | 3302 | 142 | 4 | 94 | 66 | 48 | 3 |
| **All available data (including women without HIV and/or women referred from other health facilities)** | | | | | | | | |
| **Central Africa** |  |  |  |  |  |  |  |  |
| Busanza HC | 2020 | 318 | - |  | - |  | - |  |
| Gikondo HC | 2018 | 1146 | 20 | 2 | - |  | 3 | 15 |
| Masaka HC | 2018 | 558 | - |  | - |  | - |  |
| Nyarugunga HC | 2019 | 367 | 2 |  | - |  | 2 | 100 |
| **East Africa** |  |  |  |  |  |  |  |  |
| MOI Teaching And Referral Hospital | 2018 | 5174 | 5174 | 100 | 4865 | 94.0 | 308 | 6 |
| Lumumba hospital | 2018 | 4721 | 0 |  | 0 |  | 0 |  |
| Morogoro hospital | 2020 | 2421 | 926 | 38 | - |  | 57 | 6 |
| Masaka Regional Referral Hospital | 2019 | 8931 | 46 | 1 | 0 |  | 5 | 11 |
| **Southern Africa** |  |  |  |  |  |  |  |  |
| Rahima Moosa MCH | 2020 | 271 | - |  | - |  | - |  |
| Chongwe rural HC | 2020 | 286 | 1510 | h | 1093 | 72.4 | 99 | 7 |
| Chiure Hospital | - | - | 293 | - | - |  | 33 | 13 |
| Ngwerere rural HC | 2020 | 524 | 346 | 66 | 409 | h | 12 | 2 |
| **West Africa** |  |  |  |  |  |  |  |  |
| CIRBA | 2018 | 1674 | 251 | 15 | 251 | 100 | 0 | 0 |
| Nigerian Institute Of Medical Research | 2017 | 5567 | 1449 | 26 | 1264 | 87 | 180 | 12 |
| USAC | 2016 | 1988 | 409 | 20 | 399 | 98 | 10 | 2 |

†Year cervical cancer screening was started: South Africa (1975), Nigeria (2004), Zambia (2006), Kenya (2007/2008), Uganda (2009),

Côte d’Ivoire (2009), Tanzania (2010-2017), Mozambique (2011) Zimbabwe (2011) Malawi (2012) Rwanda (2016/2019) Burundi (2017) Burkina Faso (2019)

Abbreviations: HC, Health centre ; CEPREF, « Centre d'Excellence de Prise en charge des patients du VIH/SIDA », CNTS, « Centre National de Transfusion Sanguine »,  CIRBA, « Centre Intégré de Recherches Biocliniques d'Abidjan », USAC, «Unité de Soins Ambulatoires et de Conseil » ; MCH, Mother and Child Hospital

^a^Number screened/Number of women in care, ^b^Number screened negative/Number screened, ^c^Number screened positive/Number screened, ^d^Number screened for first time/Number screend, ^e^Number screened positive for first time/Number screened, ^f^Number with inconclusive results/Number screened, ^g^percentage greater than 100 (528) due to referrals for screening, ^g,h^Percentage greater than 100 (118) due to referrals for screening

**Supplement Table 8: Treatment of pre-cancerous lesions: rates according to changing denominators**

| **Region and Facility name** | **Screen positive** | | | **Treated** | | **Treatment postponed** | | **Postponed treatment received** | | **Referred for treatment** | | **Received treatment after referral** | | **Post treatment complications** | | **Precancer cure rate** | |
| --- | --- | --- | --- | --- | --- | --- | --- | --- | --- | --- | --- | --- | --- | --- | --- | --- | --- |
|  | **n** | | Rate [%]^a^ | **n** | Rate [%]^b^ | **n** | Rate [%]^c^ | **n** | Rate [%]^d^ | **n** | Rate [%]^e^ | **n** | Rate [%]^f^ | **n** | Rate [%]^g^ | **n** | Rate [%]^h^ |
| **Data for WLHIV in care** | | | | | | | | | | | | | | | | | |
| **East Africa** |  |  | |  |  |  |  |  |  |  |  |  |  |  |  |  |  |
| Tumbi Regional Referral Hospital | 5 | 1.2 | | - | - | - | - | - | - | - |  | - | - | - |  | - | - |
| Infectious Diseases Institute | 60 | 11 | | - | - | - | - | - | - | 40 | 67 | - | - | - |  | - | - |
| **Southern Africa** |  |  | |  |  |  |  |  |  |  |  |  |  |  |  |  |  |
| Lighthouse Trust | 124 | 2.5 | | 58 | 47 | - | - | - | - | - |  | - | - | - |  | - | - |
| Chiure Hospital | 33 | 13 | | - | - | - | - | - | - | - |  | - | - | - |  | - | - |
| Kanyama | 1040 | 23 | | 143 | 14 | 0 | 0 | 0 | 0 | 118 | 11 | 0 | 0 | 0 | 0 | 1 | 0.7 |
| George Health Centre | 136 | 3 | | 136 | 100 | 4 | 3 | 4 | 100 | 59 | 43 | 59 | 100 | 0 | 0 | 15 | 11 |
| Newlands Clinic | 276 | 9 | | 268 | 97 | 18 | 7 | 18 | 100 | 258 | 94 | 244 | 95 | 0 | 0 | 167 | 62 |
| **West Africa** |  |  | |  |  |  |  |  |  |  |  |  |  |  |  |  |  |
| CEPREF Yopougon | 111 | 22.9 | | 85 | 77 | 5 | 4.5 | 5 | 100 | 26 | 23 | - | - | 0 | 0 | 106 | i |
| CNTS- Public-Ko'khoua | 28 | 52 | | - | - | - |  | - | - | - |  | - | - | - |  | - | - |
| Hôpital De Jour Du CHU Souro Sanou | 48 | 34 | | 24 | 50 | 6 | 13 | - | - | 6 | 13 | - | - | 0 | 0 | - | - |
| **All available data (including women without HIV and women referred from other health facilities)** | | | | | | | | | | | | | | | | | |
| **Central Africa** |  |  | |  |  |  |  |  |  |  |  |  |  |  |  |  |  |
| Gikondo HC | 3 | 15 | | - | - | - | - | - | - | - | - | - | - | - | - | - | - |
| Nyarugunga HC | 2 | 100 | | - | - | - | - | - | - | - | - | - | - | - | - | - | - |
| **East Africa** |  |  | |  |  |  |  |  |  |  |  |  |  |  |  |  |  |
| Moi Teaching and Referral Hospital | 308 | 88 | | 45 | 15 | 0 | 0 | 0 | 0 | 0 | 0 | 0 | 0 | 3 | 7 | 42 | 93 |
| Lumumba Hospital | 0 | 0 | | 0 | 0 | 0 |  | 0 | 0 | 0 | 0 | 0 | 0 | 0 | 0 | 0 | 0 |
| Hospital | 57 | 6 | | - | - | - |  | 1 | - | 0 | 0 | 0 | 0 | 0 | 0 | 0 | 0 |
| Masaka Regional Referral Hospital | 5 | 11 | | 0 | 0 | 0 | 0 | 0 | 0 | 0 | 0 | 0 | 0 | 0 | 0 | 0 | 0 |
| **Southern Africa** |  |  | |  |  |  |  |  |  |  |  |  |  |  |  |  |  |
| Chongwe rural HC | 99 | 7 | | 12 | 12 | - | - | 0 | 0 | 67 | 68 | 0 | 0 | 0 | 0 | 0 | 0 |
| Ngwerere Rural Health Centre | 69 | 15 | | 11 | 16 | 0 | 0 | - | - | 1 | 2 | 0 | 0 | - | - | 4 | 36 |
| CIRBA | 7 | 3 | | 0 | 0 | - | - | - | - | - | - | - | - | - | - | - | - |
| **West Africa** |  |  | |  |  |  |  |  |  |  |  |  |  |  |  |  |  |
| Nigerian Institute Of Medical Research | 180 | 12 | | - | - | 3 | 2 | 0 | 0 | 180 | 100 | - | - | - |  | - | - |
| USAC | 10 | 2 | | 10 | 100 | 0 | 0 | - | 0 | 10 | 100 | 8 | 80 | 0 | 0 | 8 | 80 |

Abbreviations: HC, Health centre ; CEPREF, « Centre d'Excellence de Prise en charge des patients du VIH/SIDA », CNTS, « Centre National de Transfusion Sanguine », CIRBA, « Centre Intégré de Recherches Biocliniques d'Abidjan », USAC, «Unité de Soins Ambulatoires et de Conseil » ; CHU, « Centre Hospitalier Universitaire »

^a^Number screened positive/Number of women in care, ^b^Number treated/Number screened positive, ^c^Number with treatement postponed/Number screened positive, ^d^Number who received treatment after being postponed/Number with treatment postponed, ^e^Number referred for treatment/Number screened positive, ^f^Number who received treatement after refferral/Number referred for treatment, ^g^Number with post treatment complications/Number treated , ^h^Number free from precaner at follow-up/Number treated, ^i^Percentage greater than 100 (124.7) due to referrals for post-treatment follow-up.

**Supplement Table 9: Cervical cancer diagnosis and management**

| **Region and Facility Name** | **Screen positive** | | **Suspicious CC** | | **Diagnosed for CC** | | **Confirmed CC** | | **CC Management** | |
| --- | --- | --- | --- | --- | --- | --- | --- | --- | --- | --- |
| **Data for WLHIV in care** | | | | | | | | | | |
|  | **n** | Rate [%]^a^ | **n** | Rate [%]^b^ | **n** | Rate [%]^c^ | **n** | Rate [%]^d^ | **n** | Rate [%]^e^ |
| **East Africa** |  |  |  |  |  |  |  |  |  |  |
| Tumbi Regional Referral Hospital | 5 | 1 | - | - | - | - | - | - | 0 | 0 |
| Infectious Diseases Institute | 60 | 11 | 0 | - | 2 | - | 0 | - | 0 | 0 |
| **Southern Africa** |  |  |  |  |  |  |  |  |  |  |
| Chiure Hospital | 33 | 13 | - | - | - | - | - | - | - | - |
| Kanyama | 1040 | 23 | 10 | 1 | 0 | 0 | 0 | - | 0 | - |
| George Health Centre | 136 | 4 | 20 | 15 | 0 | 0 | 0 | - | 0 | - |
| Newlands Clinic | 276 | 9 | 4 | 44 | 5 | f | 3 | 75 | 3 | 100 |
| Lighthouse Trust | 124 | 2.5 | 17 | 14 | - | - | - | - | - | - |
| **West Africa** |  |  |  |  |  |  |  |  |  |  |
| CEPREF Yopougon | 111 | 22.9 | 3 | 2.7 | 3 | 100 | 3 | 100 | - | - |
| CNTS - Public-Ko'khoua | 28 | 52 | - | - | - | - | - | - | - | - |
| Hôpital de Jour du CHU Souro Sanou | 48 | 33.8 | 2 | 4 | 48 | 100 | 1 | 6 | 1 | 100 |
| **All available data (including women without HIV and women referred from other health facilities)** | | | | | | | | | | |
| **Central Africa** |  |  |  |  |  |  |  |  |  |  |
| Gikondo Health Center | 3 | 15 | - | - | - | - | - | - | - | - |
| Nyarugunga Health Center | 2 | 100 | - | - | - | - | - | - | - | - |
| **East Africa** |  |  |  |  |  |  |  |  |  |  |
| Moi Teaching And Referral Hospital | 308 | 88 | 164 | 53 | 193 | g | 141 | 73 | 342 | h |
| Lumumba Hospital | 0 | 0 | 0 | 0 | 0 | - | 0 | 0 | 0 | 0 |
| Morogoro Hospital | 57 | 6 | 0 | 0 | 0 | - | 0 | 0 | 0 | 0 |
| Masaka Regional Referral Hospital | 5 | 11 | 0 | 0 | 0 | - | 0 | - | 0 | 0 |
| **Southern Africa** |  |  |  |  |  |  |  |  |  |  |
| Chongwe Rural HC | 99 | 7 | 8 | 8 | 0 | - | 0 | - | 0 | 0 |
| Ngwerere Rural Health Centre | 69 | 15 | 1 | 8 | 0 | - | 0 | - | - | - |
| Newlands Clinic | 276 | 9 | 4 | 1 | 5 | i | 3 | 75 | 3 | 100 |
| **West Africa** |  |  |  |  |  |  |  |  |  |  |
| CIRBA | 7 | 3 | - | - | - | - | 0 | - | - | - |
| Nigerian Institute of Medical Research | 180 | 12 | 5 | 3 | 29 | j | 2 | 50 | 4 | 200 |
| USAC | 10 | 2 | 2 | 20 | - | - | - | - | 0 | 0 |

Abbreviations: HC, Health centre ; CEPREF, « Centre d'Excellence de Prise en charge des patients du VIH/SIDA », CNTS, « Centre National de Transfusion Sanguine », CIRBA, « Centre Intégré de Recherches Biocliniques d'Abidjan », USAC, «Unité de Soins Ambulatoires et de Conseil » ; CHU, « Centre Hospitalier Universitaire » ; CC, Cervical Cancer

^a^Number screened positive/Number of women in care, ^b^Number with suspected CC/Number screened positive, ^c^Number with diagnosis of CC performed/Number with suspected CC, ^d^Number with confirmed CC/Number with diagnosis performed for CC, ^e^Number with ICC managed/Number with confirmed CC, ^f^Percentage greater than 100 (118) due to referrals for CC diagnosis, ^g,h,i,j^ Rate higher than 100% due to referrals for CC diagnosis and management from other sites.

**Supplement Table 10: Referral for diagnosis and treatment of cervical cancer**

| **Region and Facility Name** | **Screen positive** | | **Suspected CC** | | **Referred for suspected cancer** | | **Diagnosed after referral** | | **Treated after referral** | |
| --- | --- | --- | --- | --- | --- | --- | --- | --- | --- | --- |
|  | **n** | Rate [%]^a^ | **n** | Rate [%]^b^ | **n** | Rate [%]^c^ | **n** | Rate [%]^d^ | **n** | Rate [%]^e^ |
| **East Africa** |  |  |  |  |  |  |  |  |  |  |
| Moi Teaching and Referral Hospital | 308 | 88 | 164 | 53 | 0 | 0 | 0 | 0 | 0 | 0 |
| Lumumba hospital | 0 | 0 | 0 | 0 | 0 | 0 | 0 | 0 | 0 | 0 |
| Morogoro Hospital | 57 | 6 | 0 | 0 | 7 | - | 0 | 0 | 0 | 0 |
| Infectious Diseases Institute | 60 | 11 | 0 | 0 | - | - | - | - | - | - |
| Masaka Regional Referral Hospital | 5 | 11 | 0 | 0 | 0 | 0 | 0 | 0 | 0 | 0 |
| **Southern Africa** |  |  |  |  |  |  |  |  |  |  |
| Lighthouse Trust Martin Preuss Center | 124 | 3 | 17 | 14 | - | - | - | - | - | - |
| Chiure hospital | 33 | 13 | - | - | - | - | - | - | - | - |
| Chongwe Rural HC | 99 | 7 | 8 | 8 | 8 | 100 | 0 | 0 | 0 | 0 |
| Kanyama | 1040 | 23 | 10 | 1 | 10 | 100 | 0 | 0 | 0 | 0 |
| Ngwerere rural health centre | 69 | 15 | 1 | 8 | 1 | 100 | 0 | 0 | 0 | 0 |
| George health centre | 136 | 4 | 20 | 15 | 20 | 100 | 0 | 0 | 0 | 0 |
| Newlands Clinic | 276 | 9 | 4 | 1 | 4 | 100 | 2 | 50 | 1 | 50 |
| **West Africa** |  |  |  |  |  |  |  |  |  |  |
| CIRBA | 7 | 3 | - | - | - |  | - | - | - | - |
| CEPREF Yopougon | 111 | 23 | 3 | 3 | 3 | 100 | 3 | 100 | - | - |
| Institute of Medical Research, Lagos, Nigeria | 180 | 12 | 5 | 3 | 2 | 40 |  |  |  |  |
| Hôpital de Jour du CHU Souro Sanou | 48 | 34 | 2 | 4 | 2 | 100 | 1 | 50 | 1 | 100 |
| USAC | 10 | 2 | 2 | 20 | 2 | 100 |  |  |  |  |

Abbreviations: HC, Health centre ; CEPREF , « Centre d'Excellence de Prise en charge des patients du VIH/SIDA », CIRBA, « Centre Intégré de Recherches Biocliniques d'Abidjan », USAC, «Unité de Soins Ambulatoires et de Conseil » ; CHU, « Centre Hospitalier Universitaire » ; ICC, Invasive Cervical Cancer

^a^Number screened positive/Number of women in care, ^b^Number with suspected CC/Number screened positive, ^c^Number with suspected CC referred/Number with suspected CC, ^d^Number diagnosed with CC after referral/Number with suspected CC referred, ^e^Number with CC managed after referral/Number diagnosed with CC after referral.

**Supplement Table 11: Number of women screened by type of test**

| **Region and Facility Name** | **# screened VIA** | **Screen-positive VIA** | **# screened VIAC** | **Screen-positive VIAC** | **# screened VILI** | **Screen-positive VILI** | **# screened Pap** | **Screen-positive Pap** | **# screened HPV DNA** | **Screen-positive HPV DNA** |
| --- | --- | --- | --- | --- | --- | --- | --- | --- | --- | --- |
| **Data for WLHIV in care** | | | | | | | | | | |
| **East Africa** |  |  |  |  |  |  |  |  |  |  |
| Tumbi Regional Referral Hospital | 422 | 5 | 0 | 5 | 0 | 0 | 0 | 0 | 0 | 0 |
| Infectious Diseases Institute | 548 | 63 | 0 | 0 | 0 | 0 | 0 | 0 | 0 | 0 |
| **Southern Africa** |  |  |  |  |  |  |  |  |  |  |
| Lighthouse Trust | 4881 | 124 | - | - | - | - | - | - | 253 | - |
| Chiure hospital | 293 | 33 | 0 | 0 | 0 | 0 | 0 | 0 | 0 | 0 |
| Kanyama | 2504 | 152 | 0 | 0 | 0 | 0 | 0 | 0 | - | 1934 |
| George health centre | 3731 | 156 | 156 | 156 | 0 | 0 | 0 | 0 | 19 | 1 |
| **West Africa** |  |  |  |  |  |  |  |  |  |  |
| CEPREF Yopougon | 482 | 114 | 485 | 114 | 111 | 114 | 0 | 0 | 0 | 0 |
| CNTS - Public-Ko'khoua | 702 | 28 | 702 | 28 | 702 | 28 | - | - | - | - |
| Hopital de Jour CHU Souro Sanou | 47 | 25 | 47 | 25 | 47 | 25 | 0 | 0 | 142 | 48 |
| **All available data (including women without HIV and women referred from other health facilities)** | | | | | | | | | | |
| **Central Africa** | | | | | | | | | | |
| Gikondo HC | 20 | 3 | - | - | - | - | - | - | - | - |
| Nyarugunga HC | 2 | 2 | 226 | 2 | - | - | - | - | - | - |
| **East Africa** | | | | | | | | | | |
| MOI Teaching and Referral Hospital | 5174 | 308 | 0 | 0 | 0 | 0 | 28 |  | 0 | 0 |
| Morogoro Hospital | 926 | 57 | 0 | 0 | 0 | 0 | 0 | 0 | 0 | 0 |
| Masaka Regional Referral Hospital | 46 | 5 | 0 | 0 | 0 | 0 | 4 | 4 | 0 | 0 |
| **Southern Africa** | | | | | | | | | | |
| Chongwe Rural HC | 864 | 99 | 864 | 99 | 0 | 0 | 0 | 0 | 646 | 296 |
| Ngwerere rural HC | 346 | 69 | 346 | 12 | - | - | - | - | 131 | 57 |
| Newlands Clinic | 0 | 0 | 2807 | 272 | 0 | 0 | 95 | 5 | 22 | 1 |
| **West Africa** | | | | | | | | | | |
| CIRBA | 251 | 7 | 251 | 7 | - | - | - | - | - | - |
| Department of Clinical Science, Nigerian Institute of Medical Research | 933 | 102 | 0 | 0 | 709 | 113 | 71 | 9 | 235 | 46 |
| USAC | 409 | 10 | - | - | - | - | 7 | - | - | - |

Abbreviations: VIA, Visual Inspection with Acetic acid ; VIAC, Visual Inspection with Acetic acid and cervicography ; VILI, Visula Inspection with Lugol’s Iodine ; Pap, Papanicolaou test ; HPV DNA, Human Papillomavirus DeoxyriboNucleic acid test, WLHIV, Women Living with HIV, HC, Health centre ; CEPREF , « Centre d'Excellence de Prise en charge des patients du VIH/SIDA », CNTS, « Centre National de Transfusion Sanguine »,  CIRBA, « Centre Intégré de Recherches Biocliniques d'Abidjan », USAC, «Unité de Soins Ambulatoires et de Conseil » ; CHU, « Centre Hospitalier Universitaire »

**Supplement Table 12: List of sites by region and country**

| **Central Africa** | **East Africa** | **West Africa** | **Southern Africa** |
| --- | --- | --- | --- |
| Site - ***Country*** | Site - ***Country*** | Site - ***Country*** | Site - ***Country*** |
| Centre Hospitalo-Universitaire de Kamenge (CHUK) – ***Burundi*** | Moi Teaching and referral Hospital- ***Kenya*** | Centre Intégré de Recherches Biocliniques (CIRBA) *Pédiatrique et Adulte* ***– Cote d’Ivoire*** | Lighthouse Trust ***– Malawi*** |
| L’Association Nationale de Soutien aux Seropositifs et maladies du sida (ANSS) - ***Burundi*** | Lumumba Hospital ***– Kenya*** | Centre National de Transfusion Sanguine (CNTS)***– Cote d’Ivoire*** | Rahima Moosa mother and child hospital***– South Africa*** |
| Nyarugunga health centre – ***Rwanda*** | Morogoro Regional Hospital – ***Tanzania*** | Centre de Prise en charge, de Recherche et de Formation (CEPREF) ***- Cote d’Ivoire*** | Newlands clinic ***– Zimbabwe*** |
| Gidondo health centre – ***Rwanda*** | Tumbi Special Hospital ***– Tanzania*** | Unité de Soins Ambulatoire et de Conseil (USAC) ***- Cote d’Ivoire*** | Chongwe health centre ***– Zambia*** |
| Kabuga health centre– ***Rwanda*** | Kisesa Health Centre ***– Tanzania*** | Centre Hospitalier Universitaire de Sourô Sanou *–* ***Burkina Faso*** | George health centre***– Zambia*** |
| Busanza health centre – ***Rwanda*** | Mbarara Regional Hospital ***– Uganda*** | Department of Clinical Science, Nigerian Institute of Medical Research, Lagos- ***Nigeria*** | Kanyama hospital ***– Zambia*** |
| Masaka health centre– ***Rwanda*** | Masaka Regional Hospital ***– Uganda*** |  | Ngwerere health centre ***– Zambia*** |
|  | Infectious Diseases Institute ***– Uganda*** |  | Seboche Mission hospital ***– Lesotho*** |
|  |  |  | Chiure hospital ***– Mozambique*** |
| **7 sites** | **8 sites** | **6 sites** | **9 sites** |

Supplement 13: **Good practices identified in sites visited**

During our study, we performed sites visits mainly in southern Africa and recorded some good practices across items from screening organisation to data reporting. Regarding screening organisation and costs, three sites had a dedicated unit for screening with dedicated staff and infrastructure for screening and pre-cancer treatment. This strategy reduced some previously identified barriers to screening including long patient waiting times by eliminating multitasking of staff across units. This also improved patient-provider communication, thus efficiency across the screening pathway. Cervical cancer screening and treatment of pre-cancerous lesions were free of charge in all sites that had treatment services on-site. This improved accessibility of these services to women who were unable to pay for these services. Task shifting was common. In all ten sites visited in SSA, trained nurses performed screening with VIA/VILI and VIAC, and treatment of lesions using cryotherapy or thermal ablation. In one site, the capacity of three laboratory technicians had been enhanced to process slides for pathology. This eased bottlenecks in services delivery mostly linked to high workload on physicians. In southern Africa, two research centres had created unique patient identifiers. One of the centres (Centre for Infectious Disease Epidemiology and Research, Cape Town), assigned each patient a code at ART initiation and attached the printed codes to patient files for subsequent consultations and data collection. The other centre (the Western Cape Provincial Health Data Centre, Cape Town) used unique identifiers to link records from the fragmented databases (laboratory, pharmacy, admissions, disease codes, transfers) and infer health conditions.

In two other sites, weekly and monthly reports for CC screening were produced by CC screening staff, and transmitted to the Ministry of Health which allowed for programme monitoring. A few sites had created partnerships with the U.S President’s Emergency Plan for AIS Relief (PEPFAR), Ariel Glaser foundation, Agence Nationale de Recherche sur le Sida et les hépatitis virales (ANRS) and the Ruedi Lüthy foundation, who provided some screening infrastructure, contributed to training staff on screening and treatment and supported the development of electronic data systems for data collection and monitoring. Partnerships has been reported by IARC as one of the best practices in cervical screening programmes [1].

1. Best practices in cervical screening programmes: audit of cancers, legal and ethical frameworks, communication, and workforce competencies. Lyon, France: International Agency for Research on Cancer (IARC Working Group Reports, No. 11).
